# Septoria tritici blotch resistance gene *Stb15* encodes a lectin receptor-like kinase

**DOI:** 10.1038/s41477-025-01920-2

**Published:** 2025-03-14

**Authors:** Amber N. Hafeez, Laetitia Chartrain, Cong Feng, Florence Cambon, Martha Clarke, Simon Griffiths, Sadiye Hayta, Mei Jiang, Beat Keller, Rachel Kirby, Markus C. Kolodziej, Oliver R. Powell, Mark A. Smedley, Burkhard Steuernagel, Wenfei Xian, Luzie U. Wingen, Shifeng Cheng, Cyrille Saintenac, Brande B. H. Wulff, James K. M. Brown

**Affiliations:** 1 John Innes Centre, Norwich Research Park, Norwich, UK; 2 Plant Science Program, Biological and Environmental Science and Engineering Division, King Abdullah University of Science and Technology, Thuwal, Saudi Arabia; 3 Agricultural Genomics Institute at Shenzhen, Chinese Academy of Agricultural Science, Shenzhen, China; 4 Université Clermont Auvergne, INRAE, GDEC, Clermont-Ferrand, France; 5 Department of Plant and Microbial Biology, University of Zürich, Zurich, Switzerland; 6 Present Address: Plant Science Program, Biological and Environmental Science and Engineering Division, King Abdullah University of Science and Technology, Thuwal, Saudi Arabia; 7 Present Address: School of Life Sciences Weihenstephan, Technische Universität München, Freising, Germany

**Keywords:** Pattern recognition receptors in plants, Genome-wide association studies, Transgenic plants

## Abstract

Septoria tritici blotch (STB), caused by the Dothideomycete fungus *Zymoseptoria tritici*, is one of the most damaging diseases of bread wheat (*Triticum aestivum*)^1^ and the target of costly fungicide applications^2^. In line with the fungus’s apoplastic lifestyle, STB resistance genes isolated to date encode receptor-like kinases (RLKs) including a wall-associated kinase (*Stb6*) and a cysteine-rich kinase (*Stb16q*)^3,4^. Here we used genome-wide association studies on a diverse panel of 300 whole-genome shotgun-sequenced wheat landraces (WatSeq consortium^5^) to identify a 99-kb region containing six candidates for the *Stb15* resistance gene. Mutagenesis and transgenesis confirmed a gene encoding an intronless G-type lectin RLK as *Stb15*. The characterization of *Stb15* exemplifies the unexpected diversity of RLKs conferring *Z. tritici* resistance in wheat.

## Abstract (web summary)

*Stb15* provides resistance to Septoria tritici blotch in wheat and encodes a G-type lectin receptor-like kinase. The three cloned *Stb* genes, which are effective against different pathogen isolates, encode diverse receptor-like kinases with extracellular domains potentially involved in sugar binding.

## Main

The domestication of wheat 10,000 years ago heralded the dawn of modern agriculture in western Eurasia while providing an opportunity for the specialization of an uninvited guest: the fungal pathogen *Zymoseptoria tritici*^6^. Understanding and bolstering genetic resistance to this pathogen could aid in reclaiming ~24 million tonnes of wheat yield lost to Septoria tritici blotch (STB) each year^1,7^.

During its interaction with wheat, *Z. tritici* colonizes the apoplast through the stomata and commences a period of asymptomatic growth wherein effectors are released: molecules that suppress host defences or make the host amenable to colonization^8^. Host resistance proteins may directly or indirectly recognize these effectors and modulate defence responses, described in apoplastic interactions as effector-triggered defence or the “invasion model”^9–11^. If undetected, the pathogen switches to its necrotrophic life stage, resulting in the release of host nutrients and the rapid growth and proliferation of the pathogen^12^. Symptoms ultimately manifest as necrotic lesions on the leaf surface containing pycnidia (asexual fruiting bodies), which produce conidia that may disperse up to one metre by rain splash, allowing further cycles of colonization and thus quick progress of the disease^13^.

Twenty-three major genes controlling isolate-specific resistance to STB (*Stb* genes) have been mapped in wheat^14–16^, but *Stb* gene cloning has lagged behind efforts for other wheat diseases. *Stb6* on chromosome 3AS, conferring race-specific resistance to *Z. tritici*^3,17^, encodes a wall-associated receptor kinase, a subfamily within the receptor-like kinase (RLK) family in plants, with a galacturonan-binding domain^3^. *Stb16q* on chromosome 3D^18^ encodes a cysteine-rich receptor kinase with two DUF26 domains^4^. *Stb15* is a major gene for resistance to *Z. tritici* isolate IPO88004, mapped to a 36 cM region in the cultivar Arina^19^*. Stb15* is a good candidate for cloning and thus for studying the biology of STB resistance owing to its large phenotypic effect resulting in full resistance to avirulent isolates, which is rare among *Stb* genes^14^, and because it is widely present throughout European wheat cultivars (although it no longer confers field resistance in the United Kingdom^20^ and possibly elsewhere).

Here we apply genome-wide association studies (GWAS) to map resistance to STB in the diverse Watkins collection of pre-Green Revolution wheat landraces, which provides a unique opportunity to study interactions with *Z. tritici* in a well-adapted yet highly genetically diverse context^21–23^. This panel represents the genetic and phenotypic variability of wheat before the Green Revolution, primarily throughout its pre-Columbian range across Eurasia and North Africa. Whole-genome shotgun sequencing of a core collection of 300 Watkins lines, selected to maximize genetic representation, offers access to the entire genetic diversity of the panel and permits mapping of resistance genes at major quantitative trait loci for use in research and breeding^5^.

**Fig. 1:**
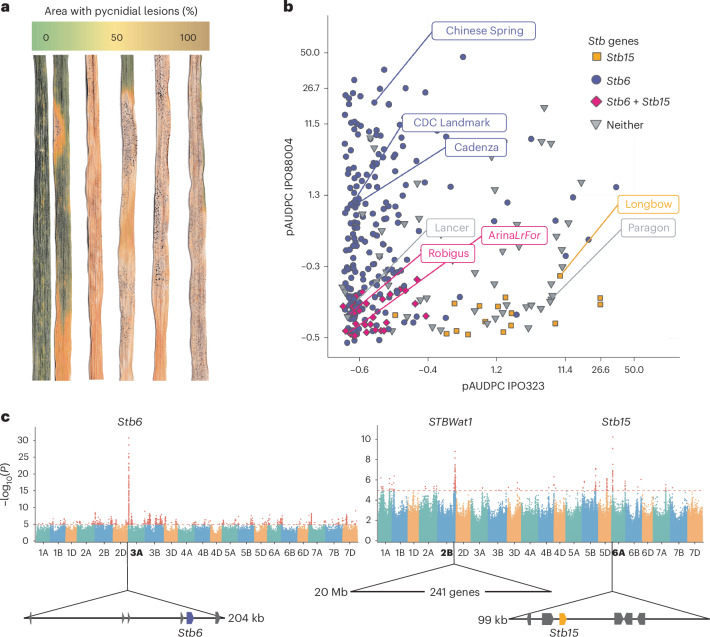
Race-specific resistance to *Z. tritici* in the wheat Watkins landrace panel associates with discrete disequilibrium blocks. **a**, Quantitative variation in pycnidia and necrosis phenotypes. Pictured are leaves arranged by pycnidial coverage (0–100%). **b**, Effects of *Stb6* and *Stb15* on pycnidia scores with *Z. tritici* isolates IPO88004 and IPO323. The logit pycnidial area under the disease progress curve (pAUDPC) values of the axes are back-transformed to give pAUDPC (0–100%). **c**, Manhattan plots showing the association of logit pAUDPC in response to *Z. tritici* isolates IPO323 (left) and IPO88004 (right) with SNPs mapped to Chinese Spring, in terms of Wald test *P* values, not adjusted for multiple comparisons. LD blocks associated with STB resistance are drawn as arrows beneath the chromosomes (marked in bold) with the 6A *Stb15* candidate gene marked in orange and *Stb6* in purple. The large interval for the locus *STBWat1* is also shown.

STB symptoms elicited by the *Z. tritici* isolates IPO323, avirulent to *Stb6* (ref. ^17^), and IPO88004, avirulent to *Stb15* (ref. ^19^), were scored across 300 Watkins landraces (Fig. 1a). This core panel was selected to maximize genetic representation^24^. Leaf damage (necrosis and chlorosis) and pycnidial coverage are usually, but not always, correlated^25^. Both phenotypes were recorded at five or six time points for calculation of the area under the disease progress curve (AUDPC), followed by logit transformation and linear mixed modelling (Supplementary Tables 1–4).

A single nucleotide polymorphism (SNP) matrix generated from whole-genome shotgun sequencing data of wheat cultivars and landraces was mapped to Chinese Spring and employed for GWAS^5^. As a positive control to ensure the suitability of the experimental system for mapping via this method, *Stb6* was successfully restricted to a discrete genomic interval in the core Watkins panel. We screened IPO323 on the Watkins core panel and ten control cultivars (Supplementary Table 5), including Chinese Spring, which has the functional allele of *Stb6* (ref. ^3^) (Fig. 1b). An interval on chromosome 3A was associated with both leaf damage and pycnidia phenotypes (Fig. 1c (pycnidia) and Supplementary Fig. 1 (leaf damage)). SNPs in the 3A locus were highly associated with pycnidia, with a −log_10_ *P* value of almost 30. Within this region, a linkage disequilibrium (LD) block extending from 26.10 to 27.50 Mb was identified. A smaller haploblock within it was most highly associated with resistance, from 26,035,170 to 26,238,727 bp (Supplementary Fig. 2). This 203.6-kb region contained six genes, including *Stb6* (Fig. 1c).

We then proceeded to identify *Stb15* by inoculating the panel with IPO88004 and employing GWAS. Three regions were associated with STB phenotypes (Fig. 1c (pycnidia) and Supplementary Fig. 3 (leaf damage)). A locus on 6AS had the highest *P* value for both pycnidia and damage traits and spanned a 99.1-kb region between 48,550,326 and 48,599,421 bp containing six genes in Chinese Spring. Gene sequences were compared between Arina*LrFor*, which has the *Stb15* resistance phenotype, and Chinese Spring, which does not; both lines have high-quality, annotated genome sequences. This comparison, combined with the correlation of haplotypes with the responses of landraces to IPO88004, excluded five of these genes (Supplementary Table 6 and Supplementary Fig. 4). The remaining gene, TraesCS6A02G078700 (Chinese Spring)/TraesARI6A03G03215890 (Arina*LrFor*), is predicted to encode an RLK and is strongly associated with isolate-specific resistance to IPO88004 but not with *Stb6* resistance to IPO323 (Fig. 1b), so it was selected as the most likely candidate for *Stb15*.

We also observed a significant association of pycnidia cover of IPO88004 with a locus on chromosome 2B from position 755 to 775 Mb containing 241 genes. When we removed the masking effect of lines carrying *Stb15*, the significance of the 2BL resistance increased 1,000-fold (Supplementary Fig. 5). *Stb9* has previously been mapped to 2BL^26^ but is outside of this locus (at ~808 Mb)^27^, and accessions that display resistance to IPO89011, an isolate that is avirulent on *Stb9*, are not all resistant to IPO88004 (refs. ^20,28^) (Supplementary Table 7). The LD block therefore appears to be a new locus for resistance to *Z. tritici*, temporarily designated as *STBWat1*.

**Fig. 2:**
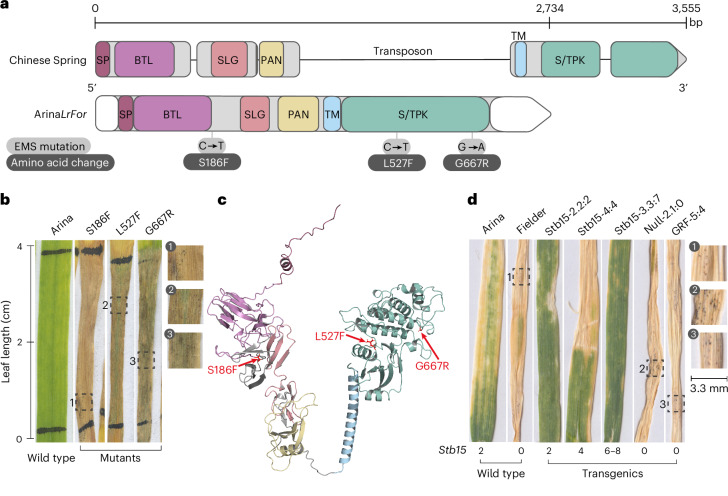
Structure and function of *Stb15.* **a**, The functional resistance allele of *Stb15* in wheat cv. Arina and Arina*LrFor* compared to the susceptible allele in cv. Chinese Spring. The predicted exons and introns are shown as rounded rectangles and lines, respectively, for Chinese Spring (RefSeq v.1.1 (ref. ^84^)) and Arina*LrFor* (Methods). Domains are highlighted: SP, signal peptide; TM, transmembrane; S/TPK, serine/threonine receptor-like protein kinase. InterProScan was used to predict protein domains, with additional adjustments made for Arina*LrFor* on the basis of the AlphaFold model (**c**). The white boxes indicate untranslated regions. The sequence variants of three EMS-induced loss-of-function mutants inoculated with *Z. tritici* isolate IPO88004 are indicated. **b**, STB phenotypes of the three EMS mutants. **c**, AlphaFold-augmented 3D structural model of Stb15. The domains are coloured as in **a**. The locations of the three EMS-induced mutations are shown in red and indicated by labelled red arrows. **d**, Cultivar Fielder stably transformed with an *Stb15* construct and inoculated with isolate IPO88004. Null-2.1:0 is a null wherein the transgene segregated out in the T_2_ family, while GRF-5:4 was transformed with the same vector backbone minus *Stb15*. Further details about the transgenic line names are provided in Supplementary Table 10. The copy number of *Stb15* is given as a fixed number or range. In **b** and **d**, the leaf sections outlined with dashed boxes and labelled 1, 2 and 3 are enlarged on the right side for improved visibility of pycnidia.

TraesARI6A03G03215890 in Arina*LrFor* was confirmed as *Stb15* by a combination of mutagenesis and transgenesis and was shown to be a lectin receptor kinase (LecRK). We screened 3,308 plants from 307 M_2_ families of an EMS-derived mutant population of cv. Arina, the donor of *Stb15* in Arina*LrFor*, for resistance to IPO88004 and identified three independent susceptible mutants (Fig. 2a,b). All three of these mutant plants had one non-synonymous transition mutation in the open reading frame of the *Stb15* candidate. The gene encodes a G-type LecRK^29^ with an intracellular serine/threonine receptor-like protein kinase and three extracellular domains: a mannose-specific bulb-type lectin (BTL), an S-locus glycoprotein (SLG) and a plasminogen/apple/nematode (PAN) domain. All three of the induced mutations resulted in replacement by larger amino acids in the BTL and kinase domains. In an AlphaFold model (Fig. 2c and Supplementary Fig. 6; predicted local distance difference test (pLDDT) score, 80.79), all three residues were in locations where mutations would be predicted to cause disruption to the protein structure. To confirm the function of the candidate gene, we synthesized a 10.9-kb genomic sequence containing 2 kb and 1.5 kb of 5′ and 3′ regulatory sequence from Arina, inserted this sequence into a binary vector and transformed wheat cv. Fielder, which is susceptible to isolate IPO88004. We obtained two independent homozygous single-copy T_2_ transgenic lines that conferred resistance to pycnidium formation by the *Stb15*-avirulent isolate IPO88004 but not IPO92006 (*Stb15*-virulent), whereas their respective nulls were susceptible to both isolates, indicating that the isolated gene sequence is sufficient to confer the *Stb15* phenotype. Transgenic lines with four copies or six to eight copies of *Stb15* were also resistant to IPO88004 relative to the controls (Fig. 2d, Supplementary Fig. 7 and Supplementary Tables 8 and 10–12). There was no evidence that *Stb15* copy number affected the resistance phenotype (Supplementary Table 10). By contrast, variation in damage was neither statistically significant nor isolate-specific (Supplementary Fig. 7c,f and Supplementary Table 9).

**Fig. 3:**
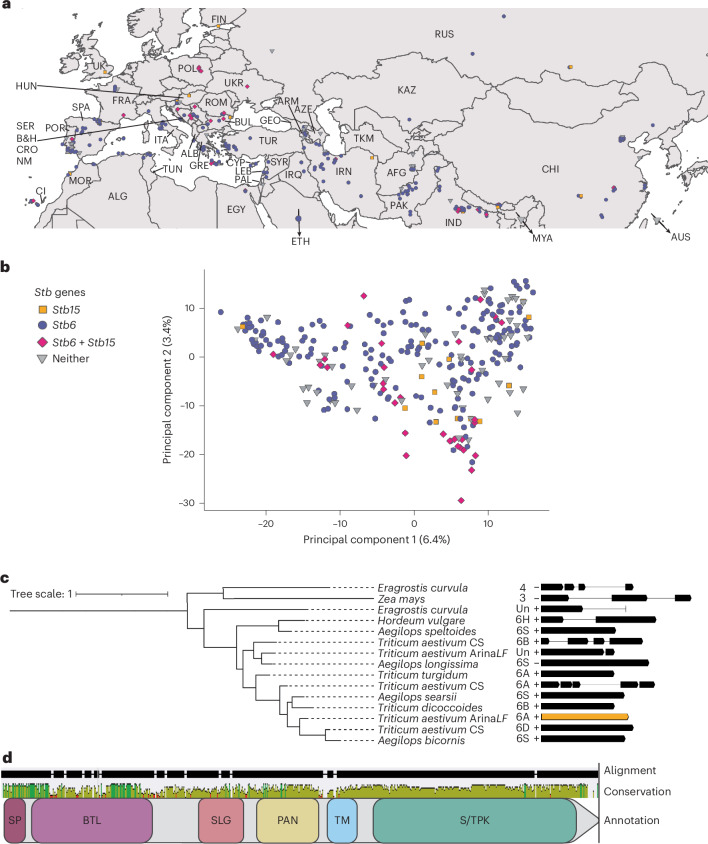
Geographic distribution and intra- and inter-species structural diversity of *Stb15.* **a**, Distribution of *Stb6* and *Stb15* in the Watkins 300 core collection. The map indicates the coordinates of local markets from which grain of landraces was obtained. Only countries from which landraces were collected are labelled. The country abbreviations are expanded in Supplementary Text 1. **b**, Principal component analysis plot of Axiom array SNPs from 300 Watkins landraces with lines containing predicted functional alleles of *Stb6* (purple), *Stb15* (orange), both (pink) or neither (grey) indicated. **c**, Maximum likelihood phylogenetic tree of proteins with homology to the Arina*LrFor* (Arina*LF*) *Stb15*-encoded allele from selected Poaceae species, including the wheat reference genome Chinese Spring (CS). The smallest non-repetitive (‘inner’) clade containing *Stb15* is shown. The intron/exon structure of *Stb15* homologues and their relative nucleotide lengths are presented (arrows indicate exon coding sequences; lines indicate introns). Species names and chromosomes are given; Un indicates homologues within scaffolds that have not yet been mapped to chromosomes. Gene IDs for homologues are given in Supplementary Table 17. **d**, Protein alignment of the homologues from **c** with alignment gaps, sequence conservation and predicted protein domains indicated. Taller, greener bars in the conservation panel indicate more conserved regions.

There are two alleles of *Stb15* in the wheat pangenome and a further two non-functional alleles in Watkins landraces (Supplementary Table 4). Alleles were defined on the basis of SNP distance (Supplementary Fig. 4 and Supplementary Tables 13 and 14). The functional Arina*LrFor* allele of *Stb15* is present across the geographic (Fig. 3a) and genetic (Fig. 3b) diversity of the Watkins core 300 collection, although it occurs in only 15% of landraces. It is often present alongside *Stb6*, which is more common (78%). A total of 14% of landraces displayed resistance to *Z. tritici*, which could not be explained by either gene. Unexplained resistance to IPO88004 (36 landraces) could be due to *STBWat1* (Fig. 1c). *Stb15* is also present in 35% of European cultivars tested using KASP markers (Supplementary Table 15).

Forty-eight proteins from 16 Poaceae species shared homology with the Arina/Arina*LrFor* Stb15 protein (Supplementary Fig. 8 and Supplementary Tables 16 and 17). Homologous genes encoding the protein sequences were found to be conserved across the Group 6 chromosomes within the Triticeae (Fig. 3c) but were also present on other chromosomes, especially Groups 3, 4 and 7 (Supplementary Fig. 8). Microsynteny of the *Stb15* mapping interval was observed in wheat lines and relatives (Supplementary Fig. 9).

We detected intron/exon structural diversity in gene annotations of proteins with homology to *Stb15* across the Poaceae, including within *Triticum aestivum* (Fig. 3c). The functional allele of *Stb15* is intronless, while in cv. Chinese Spring, lacking the *Stb15* phenotype, the gene has four introns. An intronless gene structure was also observed in gene annotations of 22 homologous proteins both within the Triticeae tribe (*Triticum, Aegilops* and *Hordeum*) and beyond it (*Brachypodium* and *Avena*), especially on Group 3 and 6D/6S chromosomes. *Stb15* clustered most closely to the Chinese Spring 6D and *Aegilops bicornis* 6S homologues, suggesting that the functional allele of *Stb15* may have originated from the D or S genomes, which share high sequence homology^30^.

Within the inner *Stb15* clade, the kinase and PAN domains were highly conserved, while the region spanning the BTL and SLG domains was variable (Fig. 3d). This suggests that this region may be under diversifying selection.

Characterization of the third *Stb* gene from a distinct subclass of the RLK protein family has the potential to enhance molecular understanding of the wheat–*Z. tritici* interaction, providing new opportunities for research and disease control. In addition, this research demonstrates the power of GWAS to greatly accelerate gene cloning for traits that are poorly understood at the molecular level. One of the factors that may limit the success of GWAS is population structure^31^. In this study, the presence of *Stb15* and *Stb6* in Watkins landraces that spanned the breadth of the genetic diversity of the panel was probably decisive to their successful mapping (Fig. 3a,b). Such wide distributions of allelic variation across the full range of relevant germplasm allow the effects of genes of interest to be separated from those of kinship. Such a distribution may be more likely for genes that were introduced early into cultivated hexaploid wheat, which appears to be the case for *Stb6*, known from both Europe and East Asia^32^. Likewise, Watkins lines with *Stb15* were obtained across the breadth of Eurasia as well as North Africa.

The diversity of intron/exon gene structures among *Stb15* homologues is unusual when compared with nucleotide-binding leucine-rich repeats, for which gene structures tend to be conserved^33^. Leucine-rich-repeat membrane-anchored proteins without intracellular kinases control resistance to the related Dothidiomycete fungus *Cladosporium fulvum*, the causal agent of leaf mould in tomato^34^, many of which^35^ share the intronless open reading frame exhibited by the functional allele of *Stb15*. Possibly, intronless gene structures have been conserved while intron gain has occurred in, for example, the Chinese Spring allele.

*Stb15* has more extracellular domains than *Stb6* or *Stb16q*, and the diversity of RLK subclasses conferring resistance is unusual compared with *Cf* genes but similar to genes conferring resistance to blackleg disease in *Brassica* species^36,37^. Equally, there are similarities shared by Stb proteins: they are transmembrane proteins with extracellular domains with a putative sugar-binding function and an intracellular kinase. Both the DUF26 domains of *Stb16q* and the G-type lectin domain of *Stb15* probably bind mannose, a building block of mannan found in cell walls of both fungi^38^ and wheat^39^. The detection of a conserved pathogen-associated molecular pattern (PAMP) fits the function of lectins, which form part of basal plant immunity and are involved in stomatal innate immunity responses in *Arabidopsis thaliana*^40^, and LecRKs confer non-host or marginal host resistance to leaf rust in barley^41^. This role could make Stb15 a target for suppression by the pathogen, or it could be part of a guard/guardee pair^42^, triggering isolate-specific resistance. Alternatively, the interaction could resemble that of the tomato receptor Cf-4 and Avr4, a passive *C. fulvum* effector that binds chitin to avoid breakdown by the host plant^34^. Another possibility is that Stb15 binds glycoproteins; AFP1 in maize was previously thought to bind chitin but in fact interacts with chitin deacetylases, most likely via their mannosylated group^43^.

LecRKs have been found to bind to secreted proteins (for example, a *Phytophthora* spp. effector^44^), which may also be the case for Stb15. A candidate gene for *AvrStb15* encoding a small secreted protein has been suggested^45^, but further work will be needed to determine the nature of its interaction with Stb15. There is thus far no evidence of a direct interaction between Stb6 and AvrStb6, also encoding a cysteine-rich small secreted protein^3,46,47^. In conclusion, our study highlights the importance of elucidating the diverse roles of *Stb* genes in defence induction for understanding the genetic basis of resistance in this economically important pathosystem.

## Methods

### Plant and pathogen material

Of 826 lines in the Watkins collection, we used a core set of 300 lines representing the majority of genetic variation in spring growth types^24^. Control lines were included in all assays, with lines selected for known responses to STB or economic importance (Supplementary Table 5). Both Arina and Arina*LrFor* were used for analyses or experiments on *Stb15* as they carry the same allele. Arina*LrFor* was derived from an Arina × Forno cross and further backcrossing with Arina^48^, where cv. Forno is susceptible to IPO88004, so the resistance in Arina*LrFor* should come from the Arina allele of *Stb15*. Seeds of Arina*LrFor* (PANG0001) are available from the Germplasm Resources Unit, John Innes Centre (JIC) (https://www.jic.ac.uk/research-impact/germplasm-resource-unit/). The *Z. tritici* isolates IPO323 (virulent on *Stb15*) and IPO88004 (virulent on *Stb6*) were used for their avirulence to *Stb6* (refs. ^3,17^) and *Stb15* (ref. ^19^), respectively. IPO323 was isolated in the Netherlands in 1981^49^ and IPO88004 in Ethiopia in 1988^50^. A third isolate, IPO90012 from Mexico^51^, was also included as a control virulent on both *Stb6* and *Stb15*; it is also avirulent on *Stb11* (ref. ^14^).

### Design and infection protocol for pathology assays at the JIC

An alpha lattice design^52^ was used for pathology experiments on 300 WatSeq landraces with five replicates per line across incomplete blocks (40-well seedling trays) in a large controlled environment room (CER) trial. This allowed the effects of tray and position in the CER to be estimated. The design was generated using Gendex (http://designcomputing.net/gendex/).

Standard pathology methods were used for the inoculation of wheat seedlings (ref. ^53^, following ref. ^50^). Seeds of the lines tested were pre-germinated in Petri dishes on 90 mm filter paper (Whatman International) containing 4 ml of 0.2 ppm gibberellic acid. The Petri dishes were placed in the dark at room temperature for 48 hours, then moved to the lab bench in daylight for a further 24 hours. Germinated seeds were planted in John Innes peat-based F2 compost in 40-well trays. The trays were placed in a Conviron CER with a 16-hour photoperiod: day temperature, 18 °C; night temperature, 12 °C; photosynthetic photon flux density, 350 µE m^−2^ at plant height. When the second leaf was fully expanded (Zadoks Growth Stage 12), usually around 14 days after germination, inoculum was prepared.

Sporulating cultures of *Z. tritici* were grown on potato dextrose agar plates for five to seven days under near-ultraviolet light (Snijders Micro Clima-Series Economic Lux Chamber) for 16 h per day at 18 °C. The cultures were flooded with 3 ml of sterile distilled water and scraped to release conidia. The concentration of conidial suspension was adjusted to the desired value of 10^6^ spores per ml. Conidial concentration was assessed using a Fuchs-Rosenthal counting chamber (Hawksley). Two drops of polyoxyethylene-sorbitan monolaurate (Tween-20; Sigma-Aldrich Chemie) were added per 50 ml of spore suspension.

Later-formed leaves were cut away so that only the primary seedling leaf remained. The seedlings were evenly sprayed with the spore suspension (20 ml per tray) on a turntable (made at JIC), using a Wiz Mini Air Compressor spray gun kit (Clarke Tools).

The trays were placed on matting within propagators, with two trays per propagator. This allowed the trays to be watered from underneath to prevent inoculum from washing off. The propagators were closed and covered with black plastic for dark incubation. The black plastic was removed after 48 hours, and the propagator lids were kept over the trays until seven days after inoculation to increase humidity and therefore the success of *Z. tritici* infection. New leaf growth was cut back twice per week to keep inoculated leaves alive and facilitate scoring.

The percentage of leaf area covered by pycnidia and leaf damage was scored by eye four to six times at intervals of two to five days over a period of 10 to 32 days post inoculation, depending on disease progress. Damage was defined as the combined area of necrosis and chlorosis. For imaging, leaves were mounted on A4 paper and scanned with a Canon LiDE 120 scanner at 600 DPI using the Canon IJ Scan Utility2 software. The standardized A4 image size allowed the dimensions of cropped images to be calculated using Adobe Illustrator.

### Statistical analysis of STB phenotypes

The AUDPC was calculated for each leaf by summing the areas of trapezia between pairs of scoring days on a graph of disease severity over time. The AUDPC data were analysed for the effects of line, isolate and experimental design factors using linear mixed modelling with random and fixed effects, via the package lmerTest^54^ in R v.4.2.2. If only fixed effects were involved, the native R analysis-of-variance function aov was used. *F*-tests were conducted to determine concise fixed models that explained as much of the variation in phenotype as possible. The quality of models was assessed using residual plots. Models were fitted to the percentage of the maximum possible AUDPC, but if the residual plots indicated non-normality or heteroscedasticity, AUDPC was transformed by the empirical logit transformation using the smallest possible AUDPC value as a coefficient to avoid logarithms of zero^55^. Generally, damage data were analysed on the percentage scale, whereas pycnidial coverage usually required logit transformation. Mean pycnidia and damage scores for each genotype were estimated through the R package emmeans^56^. Statistical analysis used R^57^ v.4.2.2.

### GWAS from the Watkins collection

The markers used for GWAS of the Watkins collection were ~10 Mb core SNPs generated from whole-genome shotgun sequencing of accessions and alignment to Chinese Spring. Extreme outlier values of phenotypic data were removed. We calculated the kinship matrix as the covariate using GEMMA-kin. On this basis, we performed GWAS using GEMMA (v.0.98.1), a computationally efficient method of conducting GWAS on large datasets^58^, with the command "gemma -miss 0.9 -gk -lm -k kinship.txt -maf [num]" (num: value of minor allele frequency threshold). In-house R scripts were used to visualize associations between SNPs and log pAUDPC as −log_10_ transformed *P* values from Wald statistics, which follow a *χ*^2^ distribution under the null hypothesis of no association. Multiple test corrections were not applied because the high covariance between closely linked SNPs makes such tests excessively conservative. *Stb6* and *Stb15* were identified as peaks of −log_10_(*P*) in the regions where they are known to be located^3,17,19^, and *STBWat1* was identified as a prominent new peak.

### Estimation of haplotypes/alleles of candidate genes

A Python script was written to identify haplotypes of the six candidate *Stb* genes in the 6AS locus (https://github.com/ambernhafeez/VCFparse_distance.git). The script parsed variant call format files (VCFs) from the alignment of Watkins and wheat lines to Chinese Spring (see above). This produced a matrix of SNP distance between all accessions, which was used to determine haplotype groups. The R package pheatmap^59^ (v1.0.12) was used to generate heat maps arranged in dendrograms from distance matrices, including associated phenotype data. Iterations of the VCF parsing script were run and plotted to identify the most useful variation for haplotype calling. Ultimately, the whole gene sequence was analysed (rather than, for example, exons alone). The dendrogram produced was manually analysed to estimate the number of haplotype groups present, defined by having a very similar number of SNPs different from Chinese Spring (SNP distance). Clusters were estimated using the cutree function in pheatmap; several iterations were performed to determine the number of clusters/haplotypes that were most informative, particularly for explaining phenotypes. This method was used to determine which Watkins landraces carry the functional Arina*LrFor* allele of the *Stb15* candidate as well as the functional Chinese Spring allele of *Stb6*: haplotype groups that contained the resistant accession and were associated with resistance were most likely to represent the functional allele.

### Generation of figures presenting Stb gene alleles in the Watkins collection

Figures wherein *Stb* gene alleles were plotted (Figs. 1a and 3a,b) were generated in R using ggplot2 (ref. ^60^, v.3.5.1) and cowplot^61^ (v.0.7.0). For Fig. 3a, the R package ggmap^62^ (v.3.0.0) was implemented for generating the map and plotting coordinates.

A principal component analysis was conducted in R v.4.2.2 using the package vcfR^63^ (v.1.14.0) to process the WatSeq VCF data for *Stb15*, the base R prcomp function to compute the principal component analysis and the vegan^64^ package (v.2.5-7) for further analysis. Composite main figures and illustrations were generated in Adobe Illustrator 2023.

### Identification of candidate *Stb* genes by bioinformatics

Candidate STB resistance genes were identified by selecting the most likely candidate from the genes in the LD block most highly associated with STB response. Several factors were considered, such as the SNP *P* value (for association with STB response), gene class, the presence of differential SNPs between susceptible and resistant wheat varieties, and the strength of correlation of predicted resistant haplotypes with STB responses (described above).

To further confirm the *Stb15* candidate, a second iteration of the GWAS was run with lines carrying predicted functional alleles of the candidate gene removed. This resulted in the loss of the association of the 6AS locus with resistance, implying that the lines removed did contain the 6AS resistance.

### Generation of an Arina EMS population

The generation of the Arina EMS population is described in Kolodziej et al.^48^. EMS mutagenesis of cv. Arina was performed with concentrations of 0.6% or 0.45% EMS (Sigma Aldrich). Seeds were incubated for 16 h in water at 4 °C, dried for 8 h on filter paper and incubated for 16 h with shaking at room temperature in EMS solution. After being washed three times for 30, 45 and 60 min and for another 30 min under running tap water, the seeds were pre-germinated on humid filter paper. Three thousand seeds of BC2F5-85 were mutagenized, and the pre-germinated seeds were propagated in the field. Single spikes of M_0_ plants were harvested, and M_1_ plants were grown and harvested in the field.

### Validation of the *Stb15* candidate through screening an Arina EMS population

When available, 12 seeds per M_2_ family were sown in a mixture of 1/2 blond and 1/2 brown peat mosses (Humustar soil, NPK 14-16-18, SARL Activert) and kept at 6 °C for four days. Subsequently, the plants were grown in a CER with sodium lamps (HQI-TS 250 W/D UVS FC2 FLH1; intensity, 300 µmol m^−2^ s^−1^) with 16 h, 21 °C day / 8 h, 18 °C night conditions and a relative humidity of 85%. Fourteen days after sowing, the plants were inoculated with a hand sprayer (Elyte 2, Berthoud) with *Z. tritici* isolate IPO88004. The plants were covered with plastic bags for three days before being returned to normal conditions. Visual evaluations were conducted at 21 and 28 days post-inoculation. All M_2_ plants carrying pycnidia were self-pollinated. M_3_ plants were evaluated for resistance to isolate IPO88004, following the procedure described above, except that inoculations were performed on six-centimetre sections in the middle of the second leaf using a paintbrush. Three plants per M_3_ family were inoculated in two independent experiments. The *Z. tritici* inoculum was prepared using YG and YPD media following the procedure described in Battache et al.^28^. Inoculation with concentrations of 1 × 10^6^ spores per ml and 1 × 10^7^ spores per ml, supplemented with 0.05% (v/v) Tween-20, were used for inoculating M_2_ and M_3_ plants, respectively. The *Stb15* candidate gene was sequenced from each susceptible M_3_ plant using Sanger sequencing following PCR amplification using the primer pairs Stb15F1/Stb15R1 and Stb15F3/Stb15R3 and the Phusion High-Fidelity Master Mix.

The primers were as follows:

Stb15F1: TCCTACTACTAGCCAAGCATGTC

Stb15R1: GCCATTGCCGTTAGAAACAG

Stb15F3: CTGTTCGAGGGAGGTTCCTA

Stb15R3: GTGCAAAGACCGCAGTATGT

### Design of the *Stb15* binary vector construct

A wheat transformation vector was assembled using standard Golden Gate MoClo assembly^65^ and traditional digestion and ligation cloning. The level 1 plasmids pL1P1R *Pv*UbiP::*hpt*–int::35sT selection cassette, pICH47742 L1P2 multiple cloning site and LacZ (Addgene no. 48001), and pL1P3ZmUbiP::GRF–GIF::NosT (Addgene no. 198047) were assembled into the level 2 acceptor pGoldenGreenGate-M (pGGG-M) (Addgene no. 165422) binary vector^66^ along with end linker pELE-3 (Addgene no. 48018). The resulting plasmid was named pGGG L2 PvUH GGLacZ GRF–GIF (available as Addgene no. 226630). The *Stb15* gene sequence was analysed using the software Geneious Prime v.2020.2.4 (Biomatters), and two restriction enzymes (SbfI and SacI) were chosen for digestion/ligation cloning. The sequence containing the *Stb15* gene (6,077 bp), consisting of a 1,917-bp promoter, a 136-bp 5′ untranslated region, a 2,290-bp coding sequence, a 306-bp 3′ untranslated region and a 1,404-bp terminator, was synthesized (Invitrogen, Thermo Fisher Scientific) with restriction enzyme recognition sites SbfI and SacI added to the 5′ and 3′ ends, respectively. The *Stb15* gene synthon was cloned into pGGG L2 PvUH GGLacZ GRF–GIF within the multiple cloning site using SbfI and SacI digestion/ligation. The resulting plasmid was named pGGG L2 Ta*Stb15* and was electroporated into the hypervirulent *Agrobacterium* strain AGL1 (refs. ^67,68^).

### *Agrobacterium* transformation of *T. aestivum* cv. Fielder

The wheat transformation method described here was based on an existing method^69^ with slight modifications. The construct incorporated GRF4–GIF1 technology^70^. Briefly, wheat cv. Fielder was grown in a CER under a long-day photoperiod (16 h at 600 μmol m^−2^ s^−1^ light, at 20 °C day and 16 °C night). Wheat spikes were collected ~14 days post anthesis (early milk stage GS73) when the immature embryos were 1–1.5 mm in diameter. Under aseptic conditions, immature embryos were isolated from surface-sterilized grain.

The isolated immature embryos were pretreated by centrifugation in liquid medium prior to *Agrobacterium* inoculation. The embryos were transferred to co-cultivation medium, scutellum side up, and incubated at 24 °C in the dark for three days of co-cultivation. The embryogenic axes were excised and discarded; then, the embryos were transferred to wheat callus induction (WCI) medium without selection for five days at 24 °C in the dark. After five days, the embryos were transferred to WCI containing 15 mg l^–1^ hygromycin and incubated at 24 °C in the dark. Subculturing onto fresh WCI with hygromycin selection at 15 mg l^–1^ occurred every two weeks over a five-week period. For the final, fifth week on WCI, the cultures were maintained in low-light conditions at 24 °C and then transferred onto wheat regeneration medium supplemented with 0.5 mg l^–1^ zeatin and 15 mg l^–1^ hygromycin in deep Petri dishes (90 mm diameter × 20 mm) and cultured under fluorescent light (100 μM m^−2^ s^−1^) with a 16 h photoperiod. Regenerated plantlets were transferred to De Wit culture tubes (Duchefa-Biochemie, W1607) containing rooting medium supplemented with 20 mg l^−1^ hygromycin. After approximately ten days, rooted plants were transferred to soil (John Innes cereal mix in 24-cell trays) and acclimatized^68^. The transgenic plants were maintained under the same growing conditions as donor material with a long-day photoperiod (16 h at 600 μmol m^−2^ s^−1^ light, at 20 °C day and 16 °C night). Transgenesis was confirmed and transgene copy number analysis performed on 58 independent lines using a Taqman relative quantification assay. The relative values of the hygromycin resistance gene (*hpt*) and *CO2* (Constans-like, AF490469) were compared in a multiplexed reaction using specific probes and primers^68^. Relative quantification values (ΔΔCt) were calculated to determine transgene copy number^71^.

### Experimental validation of the *Stb15* transgenics

The experiment to test the effect of the *Stb15* candidate on STB symptoms caused by the *Stb15*-avirulent isolate IPO88004 and the virulent control isolate IPO92006 was sown in 40-well seed trays with two experimental replicates per tray in a randomized design (ten replicates per line). This assay was then repeated with a new randomized design in a separate trial, giving up to 20 replicates per line for IPO88004 and 10 replicates per line for IPO92006. The conditions and infection protocol were as described above under ‘Design and infection protocol for pathology assays at the JIC*’*, except the inoculum concentration of IPO88004, which was adjusted to 10^7^ spores per ml because of the inherent partial resistance of cv. Fielder. The percentage leaf area covered by lesions containing pycnidia was analysed by linear mixed modelling as described in the section ‘Statistical analysis of STB phenotypes’ above. The random-effect model for pycnidia data was Replicate within Tray within Test, and Tray within Test and the fixed-effects model was Test × Isolate + Isolate + Isolate × Variety. For damage, the same fixed-effects model was used, but the random effect of Test within Variety was included due to its significant explanation of residual variance as determined by nested deviance tests. The R package emmeans was used to perform Tukey-honest-significant-difference-adjusted pairwise comparisons between all lines.

### Identification of intron/exon structure of *Stb15*

Prepublication access to an updated genome annotation of Arina*LrFor* based on RNAseq data was provided by A. Hall and M. Spannagl^72^. Additionally, alignments of the RNAseq data were manually checked in IGV (v.2.14.0)^73^ to confirm the intronless gene structure of *Stb15*.

### Protein structure prediction of Stb15

The protein structure encoded by the *Stb15* candidate gene in Arina was predicted using AlphaFold (v.2.3.2)^74^. The quality of the highest-confidence prediction was assessed using AlphaFold Analyser (v.2.0.0). Each protein sequence was also annotated using InterProScan^75^ (v.5.61-93.0). These annotations were visualized on the high-confidence protein structure using PyMol^76^ (v.2.5.2), and domain boundaries were manually expanded upon to include unannotated amino acids.

### KASP genotyping of European cultivars

KASP genotyping was carried out as described in Saintenac et al.^4^ on 278 European wheat cultivars. The marker sequences were as follows:

F = GAAGGTGACCAAGTTCATGCTGGTTTCAACTTGCAATATGATC

V = GAAGGTCGGAGTCAACGGATTGGTTTCAACTTGCCATATGATT

C = AGTGAACCAGGTGCCAAAAC

### Analyses of sequence evolution

#### Identification of potential *Stb15* homologues in plants

High-quality reference genome protein annotations of plant species were downloaded (Supplementary Table 16). Local BLASTp databases were generated using command-line BLAST^77^ v.2.13.0. The amino acid sequence of the functional *Stb15* allele from Arina*LrFor* was used as a query sequence for BLAST searches against protein databases of each species. The top 30 hits were recovered to ensure that no potential orthologues or paralogues were missed.

#### Protein alignment

A protein alignment was generated using MUSCLE^78^ v.3.8.31 with the default settings. Sequences were removed if they contained large (>350 amino acids) and divergent insertions that disrupted the alignment or if they contained less than two of the domains present in the Arina*LrFor* Stb15 sequence. Multiple splice variants were included if their predicted amino acid sequence varied.

#### Phylogenetic tree construction and analysis of the *Stb15* clade

ModelFinder^79^ was used to predict the best evolutionary model for the alignment (JTT + R10) implemented via IQ-TREE^80^ v.1.6.10. Branch supports were obtained with ultra-fast bootstrap (UFBoot2 (ref. ^81^)), and tree reconstruction was performed using IQ-TREE. The least-repetitive clade containing *Stb15* was extracted, and sequence conservation was analysed in Geneious v.2022.2.2. Genome annotations (GFFs) for each orthologue were used to draw gene structures in R v.4.2.2. For Fig. 3c and Supplementary Fig. 5, all exon annotations for each gene are presented within a single leaf, and splice variants were pruned from the tree. The tree image was generated using iTOL^82^ v.6.

#### Alignment for the consensus sequence of the inner *Stb15* clade

A small alignment of the inner *Stb15* clade was generated by MUSCLE within Geneious v.2022.2.2. It was noticed that the *Zea mays* homologue Zea_mays_Zm00001eb119590_P002 was in fact a tandem duplication containing two identical sequences of a protein encoding a partial BTL and full SLG, PAN and S/TPK domains. To reduce disruption of the alignment, one half of this protein sequence was retained. A screenshot of the consensus chart from the Geneious alignment was used in Fig. 3d.

#### Microsynteny analysis of *Stb15* orthologues in *T. aestivum*, *Aegilops tauschii*, *T. durum* and *Hordeum vulgare*

Genome annotation files (GFFs) were used to extract the names and coordinates of ten genes on each side of the *Stb15* homologues identified above, from the same genome assemblies (see ‘Analyses of sequence evolution’). Protein sequences were extracted from peptide files associated with the genome assemblies used or exported from Ensembl Plants, and the NCBI BLAST server was used to conduct a BLASTp search. Other proteins with high similarity to the query and the Conserved Domains results were used to identify the protein family. If informative results were not gained in this way, InterProScan was also used. If protein sequences were truncated or uninformative, a tBLASTx search of the nucleotide sequence was conducted against *T. aestivum*, *Brachypodium distachyon* and *Oryza sativa*. In some cases, such as *Triticum urartu* and *Aegilops searsii*, synteny with Arina*LrFor* could not be found for the investigated genes. This analysis was not exhaustive as it focused on a small number of genes within a small genomic region. It was noticed that a truncated protein was predicted for the Arina*LrFor* 6B orthologue of *Stb15* despite high similarity to the nucleotide sequence of *Stb15*. This is why this gene appears in the microsynteny analysis but not the phylogenetic trees of proteins with similarity to Stb15 (Fig. 3c and Supplementary Fig. 8). The figure depicting these results (Supplementary Fig. 9) was generated with Inkscape v.1.3.2.

### Reporting summary

Further information on research design is available in the Nature Portfolio Reporting Summary linked to this article.

## Supplementary information

Supplementary Information Supplementary Figs. 1–9, Tables 1–17 and Text 1.

Reporting Summary

Supplementary Tables Spreadsheets with data for Supplementary Tables 4, 11, 12 and 15–17.

## Data Availability

The SNP data^5^ are available via GrassRoots at https://opendata.earlham.ac.uk/wheat/under_license/toronto/WatSeq_2023-09-15_landrace_modern_Variation_Data/WatSeq_VCF_ChineseSpringRefSeqv1.0/. The pathology data are available via Zenodo at 10.5281/zenodo.14515753 (ref. ^83^). The *Stb15* sequence has been reported with an annotated transcript on Ensembl Plants (TraesARI6A03G03215890.1).

## Code availability

Customs scripts for analysis of STB pathology data, plot generation, haplotype analysis and principal coordinates analysis are available via GitHub at https://github.com/ambernhafeez/Stb15_Project_Analyses. AlphaFold Analyser is available via GitHub at https://github.com/Orpowell/alphafold-analyser. Software for SNP mapping and GWAS-QTL mapping^5^ and plotting is available via GitHub at https://github.com/ShifengCHENG-Laboratory/WWWG2B and https://github.com/JIC-CSB/WatSeqAnalysis/.

## References

[1] Savary, S. et al. The global burden of pathogens and pests on major food crops. *Nat. Ecol. Evol.* **3**, 430–439 (2019). 30718852 10.1038/s41559-018-0793-y

[2] O’Driscoll, A., Kildea, S., Doohan, F., Spink, J. & Mullins, E. The wheat–Septoria conflict: a new front opening up? *Trends Plant Sci.* **19**, 602–610 (2014). 24957882 10.1016/j.tplants.2014.04.011

[3] Saintenac, C. et al. Wheat receptor-kinase-like protein Stb6 controls gene-for-gene resistance to fungal pathogen *Zymoseptoria tritici*. *Nat. Genet.* **50**, 368–374 (2018). 29434355 10.1038/s41588-018-0051-x

[4] Saintenac, C. et al. A wheat cysteine-rich receptor-like kinase confers broad-spectrum resistance against Septoria tritici blotch. *Nat. Commun.* **12**, 433 (2021). 33469010 10.1038/s41467-020-20685-0 PMC7815785

[5] Cheng, S. et al. Harnessing landrace diversity empowers wheat breeding. *Nature* **632**, 823–831 (2024). 38885696 10.1038/s41586-024-07682-9 PMC11338829

[6] Stukenbrock, E. H., Banke, S., Javan-Nikkhah, M. & McDonald, B. A. Origin and domestication of the fungal wheat pathogen *Mycosphaerella graminicola* via sympatric speciation. *Mol. Biol. Evol.* **24**, 398–411 (2007). 17095534 10.1093/molbev/msl169

[7] Hafeez, A. N. et al. Creation and judicious application of a wheat resistance gene atlas. *Mol. Plant* **14**, 1053–1070 (2021). 33991673 10.1016/j.molp.2021.05.014

[8] Jones, J. D. G. & Dangl, J. L. The plant immune system. *Nature* **444**, 323–329 (2006). 17108957 10.1038/nature05286

[9] Stotz, H. U., Mitrousia, G. K., de Wit, P. J. G. M. & Fitt, B. D. L. Effector-triggered defence against apoplastic fungal pathogens. *Trends Plant Sci.* **19**, 491–500 (2014). 24856287 10.1016/j.tplants.2014.04.009 PMC4123193

[10] Cook, D. E., Mesarich, C. H. & Thomma, B. P. H. J. Understanding plant immunity as a surveillance system to detect invasion. *Annu. Rev. Phytopathol.* **53**, 541–563 (2015). 26047564 10.1146/annurev-phyto-080614-120114

[11] Kanyuka, K. & Rudd, J. J. Cell surface immune receptors: the guardians of the plant’s extracellular spaces. *Curr. Opin. Plant Biol.* **50**, 1–8 (2019). 30861483 10.1016/j.pbi.2019.02.005 PMC6731392

[12] Kema, G. H. J., Yu, D., Rijkenberg, F. H. J., Shaw, M. W. & Baayen, R. P. Histology of pathogenesis of *Mycosphaerella graminicola* in wheat. *Phytopathology* **7**, 777–786 (1996).

[13] Shaw, M. W. Assessment of upward movement of rain splash using a fluorescent tracer method and its application to the epidemiology of cereal pathogens. *Plant Pathol.* **36**, 201–213 (1987).

[14] Brown, J. K. M., Chartrain, L., Lasserre-Zuber, P. & Saintenac, C. Genetics of resistance to *Zymoseptoria tritici* and applications to wheat breeding. *Fungal Genet. Biol.* **79**, 33–41 (2015). 26092788 10.1016/j.fgb.2015.04.017 PMC4510316

[15] Yang, N., Mcdonald, M. C., Solomon, P. S. & Milgate, A. W. Genetic mapping of *Stb19*, a new resistance gene to *Zymoseptoria tritici* in wheat. *Theor. Appl. Genet.* **131**, 2765–2773 (2018). 30238255 10.1007/s00122-018-3189-0

[16] Langlands-Perry, C. et al. Resistance of the wheat cultivar ‘Renan’ to Septoria leaf blotch explained by a combination of strain specific and strain non-specific QTL mapped on an ultra-dense genetic map. *Genes* **13**, 100 (2022). 10.3390/genes13010100 PMC8774678 35052440

[17] Brading, P. A., Verstappen, E. C. P., Kema, G. H. J. & Brown, J. K. M. A gene-for-gene relationship between wheat and *Mycosphaerella graminicola*, the Septoria tritici blotch pathogen. *Phytopathology* **92**, 439–445 (2002). 18942957 10.1094/PHYTO.2002.92.4.439

[18] Ghaffary, S. M. T. et al. New broad-spectrum resistance to septoria tritici blotch derived from synthetic hexaploid wheat. *Theor. Appl. Genet.* **124**, 125–142 (2012). 21912855 10.1007/s00122-011-1692-7 PMC3249545

[19] Arraiano, L. S. et al. A gene in European wheat cultivars for resistance to an African isolate of *Mycosphaerella graminicola*. *Plant Pathol.* **56**, 73–78 (2007).

[20] Arraiano, L. S. & Brown, J. K. M. Identification of isolate-specific and partial resistance to septoria tritici blotch in 238 European wheat cultivars and breeding lines. *Plant Pathol.* **55**, 726–738 (2006).

[21] Wingen, L. U. et al. Establishing the A. E. Watkins landrace cultivar collection as a resource for systematic gene discovery in bread wheat. *Theor. Appl. Genet.* **127**, 1831–1842 (2014). 24985064 10.1007/s00122-014-2344-5 PMC4110413

[22] Winfield, M. O. et al. High-density genotyping of the A.E. Watkins Collection of hexaploid landraces identifies a large molecular diversity compared to elite bread wheat. *Plant Biotechnol. J.* **16**, 165–175 (2018). 28500796 10.1111/pbi.12757 PMC5785351

[23] Ajaz, S., Benbow, H. R., Christodoulou, T., Uauy, C. & Doohan, F. M. Evaluation of the susceptibility of modern, wild, ancestral, and mutational wheat lines to Septoria tritici blotch disease. *Plant Pathol.* **70**, 1123–1137 (2021).

[24] Arora, S. et al. A wheat kinase and immune receptor form host-specificity barriers against the blast fungus. *Nat. Plants* **9**, 385–392 (2023). 36797350 10.1038/s41477-023-01357-5 PMC10027608

[25] Kema, G. H. J. et al. Variation for virulence and resistance in the wheat–*Mycosphaerella graminicola* pathosystem I. Interactions between pathogen isolates and host cultivars. *Phytopathology* **86**, 213–220 (1996).

[26] Chartrain, L., Sourdille, P., Bernard, M. & Brown, J. K. M. Identification and location of *Stb9*, a gene for resistance to septoria tritici blotch in wheat cultivars Courtot and Tonic. *Plant Pathol.* **58**, 547–555 (2009).

[27] Amezrou, R. et al. A secreted protease-like protein in *Zymoseptoria tritici* is responsible for avirulence on *Stb9* resistance gene in wheat. *PLoS Pathog.* **19**, e1011376 (2023). 37172036 10.1371/journal.ppat.1011376 PMC10208482

[28] Battache, M. et al. Blocked at the stomatal gate, a key step of wheat Stb16q-mediated resistance to *Zymoseptoria tritici*. *Front. Plant Sci.* **13**, 921074 (2022). 35832231 10.3389/fpls.2022.921074 PMC9271956

[29] Sun, Y., Qiao, Z., Muchero, W. & Chen, J. G. Lectin receptor-like kinases: the sensor and mediator at the plant cell surface. *Front. Plant Sci.* **11**, 1989 (2020). 10.3389/fpls.2020.596301 PMC7758398 33362827

[30] Avni, R. et al. Genome sequences of three *Aegilops* species of the section Sitopsis reveal phylogenetic relationships and provide resources for wheat improvement. *Plant J.* **110**, 179–192 (2022). 34997796 10.1111/tpj.15664 PMC10138734

[31] Bartoli, C. & Roux, F. Genome-wide association studies in plant pathosystems: toward an ecological genomics approach. *Front. Plant Sci.* **8**, 763 (2017). 28588588 10.3389/fpls.2017.00763 PMC5441063

[32] Chartrain, L., Brading, P. A. & Brown, J. K. M. Presence of the *Stb6* gene for resistance to septoria tritici blotch (*Mycosphaerella graminicola*) in cultivars used in wheat-breeding programmes worldwide. *Plant Pathol.* **54**, 134–143 (2005).

[33] Steuernagel, B. et al. The NLR-Annotator tool enables annotation of the intracellular immune receptor repertoire. *Plant Physiol.*10.1104/pp.19.01273 (2020). 10.1104/pp.19.01273 PMC7271791 32184345

[34] Wulff, B. B. H., Chakrabarti, A. & Jones, D. A. Recognitional specificity and evolution in the tomato–*Cladosporium fulvum* pathosystem. *Mol. Plant Microbe Interact.* **22**, 1191–1202 (2009). 19737093 10.1094/MPMI-22-10-1191

[35] Thomas, C. M., Dixon, M. S., Parniske, M., Golstein, C. & Jones, J. D. G. Genetic and molecular analysis of tomato *Cf* genes for resistance to *Cladosporium fulvum*. *Phil. Trans. R. Soc. B* **353**, 1413–1424 (1998). 9800204 10.1098/rstb.1998.0296 PMC1692346

[36] Larkan, N. J. et al. The *Brassica napus* wall-associated kinase-like (WAKL) gene *Rlm9* provides race-specific blackleg resistance. *Plant J.* **104**, 892–900 (2020). 32794614 10.1111/tpj.14966 PMC7756564

[37] Larkan, N. J. et al. The *Brassica napus* blackleg resistance gene *LepR3* encodes a receptor-like protein triggered by the *Leptosphaeria maculans* effector AVRLM1. *New Phytol.* **197**, 595–605 (2013). 23206118 10.1111/nph.12043

[38] Miyakawa, T. et al. A secreted protein with plant-specific cysteine-rich motif functions as a mannose-binding lectin that exhibits antifungal activity. *Plant Physiol.* **166**, 766–778 (2014). 25139159 10.1104/pp.114.242636 PMC4213107

[39] Burton, R. A. & Fincher, G. B. Evolution and development of cell walls in cereal grains. *Front. Plant Sci.* **5**, 456 (2014). 25309555 10.3389/fpls.2014.00456 PMC4161051

[40] Singh, P. & Zimmerli, L. Lectin receptor kinases in plant innate immunity. *Front. Plant Sci.* **4**, 124 (2013). 23675375 10.3389/fpls.2013.00124 PMC3646242

[41] Wang, Y. et al. Orthologous receptor kinases quantitatively affect the host status of barley to leaf rust fungi. *Nat. Plants* **5**, 1129–1135 (2019). 31712760 10.1038/s41477-019-0545-2

[42] van der Hoorn, R. A. L. & Kamoun, S. From guard to decoy: a new model for perception of plant pathogen effectors. *Plant Cell* **20**, 2009–2017 (2008). 18723576 10.1105/tpc.108.060194 PMC2553620

[43] Ma, L. S. et al. Maize antifungal protein AFP1 elevates fungal chitin levels by targeting chitin deacetylases and other glycoproteins. *mBio* **14**, e00093-23 (2023). 36946727 10.1128/mbio.00093-23 PMC10128019

[44] Bouwmeester, K. et al. The lectin receptor kinase LecRK-I.9 is a novel *Phytophthora* resistance component and a potential host target for a RXLR effector. *PLoS Pathog.* **7**, e1001327 (2011). 21483488 10.1371/journal.ppat.1001327 PMC3068997

[45] Amezrou, R. et al. Quantitative pathogenicity and host adaptation in a fungal plant pathogen revealed by whole-genome sequencing. *Nat. Commun.* **15**, 1933 (2024). 38431601 10.1038/s41467-024-46191-1 PMC10908820

[46] Zhong, Z. et al. A small secreted protein in *Zymoseptoria tritici* is responsible for avirulence on wheat cultivars carrying the *Stb6* resistance gene. *New Phytol.* **214**, 619–631 (2017). 28164301 10.1111/nph.14434

[47] Kema, G. H. J. et al. Stress and sexual reproduction affect the dynamics of the wheat pathogen effector AvrStb6 and strobilurin resistance. *Nat. Genet.* **50**, 375–380 (2018). 29434356 10.1038/s41588-018-0052-9

[48] Kolodziej, M. C. et al. A membrane-bound ankyrin repeat protein confers race-specific leaf rust disease resistance in wheat. *Nat. Commun.* **12**, 956 (2021). 33574268 10.1038/s41467-020-20777-x PMC7878491

[49] Kema, G. H. J. & Van Silfhout, C. H. Genetic variation for virulence and resistance in the wheat–*Mycosphaerella graminicola* pathosystem III. Comparative seedling and adult plant experiments. *Phytopathology* **87**, 266–272 (1997). 18945169 10.1094/PHYTO.1997.87.3.266

[50] Kema, G. H. J. et al. Genetic variation for virulence and resistance in the wheat–*Mycosphaerella graminicola* pathosystem I. Interactions between pathogen isolates and host cultivars. *Phytopathology* **86**, 200–212 (1996). 10.1094/PHYTO.1997.87.3.266 18945169

[51] Kema, G. H., Sayoud, R., Annone, J. G. & Van Silfhout, C. H. Genetic variation for virulence and resistance in the wheat–*Mycosphaerella graminicola* pathosystem II. Analysis of interactions between pathogen isolates and host cultivars. *Phytopathology* **86**, 213–220 (1996). 10.1094/PHYTO.1997.87.3.266 18945169

[52] Patterson, H. D. & Williams, E. R. A new class of resolvable incomplete block designs. *Biometrika* **63**, 83–92 (1976).

[53] Arraiano, L. S., Brading, P. A. & Brown, J. K. M. A detached seedling leaf technique to study resistance to *Mycosphaerella graminicola* (anamorph *Septoria tritici*) in wheat. *Plant Pathol.* **50**, 339–346 (2001).

[54] Kuznetsova, A., Brockhoff, P. B. & Christensen, R. H. B. lmerTest package: tests in linear mixed effects models. *J. Stat. Softw.*10.18637/jss.v082.i13 (2017).

[55] McGrann, G. R. D. et al. A trade off between mlo resistance to powdery mildew and increased susceptibility of barley to a newly important disease, *Ramularia* leaf spot. *J. Exp. Bot.* **65**, 1025–1037 (2014). 24399175 10.1093/jxb/ert452 PMC3935564

[56] Lenth, R. V. et al. emmeans: Estimated marginal means, aka least-squares means. R package version 1.10 10.32614/CRAN.package.emmeans (2023).

[57] R Core Team. *R: A Language and Environment for Statistical Computing* (R Foundation for Statistical Computing, 2022).

[58] Zhou, X. & Stephens, M. Genome-wide efficient mixed-model analysis for association studies. *Nat. Genet.* **44**, 821–824 (2012). 22706312 10.1038/ng.2310 PMC3386377

[59] Kolde, R. pheatmap: Pretty heatmaps. R package version 1.0.12 (2012).

[60] Wickham, H. *Package ‘ggplot2’: Elegant Graphics for Data Analysis* (Springer, 2016).

[61] Wilke, C. O. cowplot: Streamlined plot theme and plot annotations for ‘ggplot2’. R package version 0.7.0 https://CRAN.R-project.org/package=cowplot (2016).

[62] Kahle, D. & Wickham, H. ggmap: spatial visualization with ggplot2. *R J.* **5**, 144–161 (2013).

[63] Knaus, B. J. & Grünwald, N. J. vcfr: a package to manipulate and visualize variant call format data in R. *Mol. Ecol. Resour.* **17**, 44–53 (2017). 27401132 10.1111/1755-0998.12549

[64] Oksanen, J. et al. vegan: Community ecology package. R package version 2.5-7 (2020).

[65] Werner, S., Engler, C., Weber, E., Gruetzner, R. & Marillonnet, S. Fast track assembly of multigene constructs using Golden Gate cloning and the MoClo system. *Bioeng. Bugs* **3**, 38–43 (2012). 22126803 10.4161/bbug.3.1.18223

[66] Smedley, M. A., Hayta, S., Clarke, M. & Harwood, W. A. CRISPR–Cas9 based genome editing in wheat. *Curr. Protoc.* **1**, e65 (2021). 33687760 10.1002/cpz1.65

[67] Lazo, G. R., Stein, P. A. & Ludwig, R. A. A DNA transformation-competent *Arabidopsis* genomic library in *Agrobacterium*. *Biotechnology (N. Y.)* **9**, 963–967 (1991). 1368724 10.1038/nbt1091-963

[68] Hayta, S. et al. An efficient and reproducible *Agrobacterium*-mediated transformation method for hexaploid wheat (*Triticum aestivum* L.). *Plant Methods* **15**, 152 (2019). 31867048 10.1186/s13007-019-0540-7 PMC6916513

[69] Hayta, S., Smedley, M. A., Clarke, M., Forner, M. & Harwood, W. A. An efficient *Agrobacterium*-mediated transformation protocol for hexaploid and tetraploid wheat. *Curr. Protoc.* **1**, e58 (2021). 33656289 10.1002/cpz1.58

[70] Debernardi, J. M. et al. A GRF–GIF chimeric protein improves the regeneration efficiency of transgenic plants. *Nat. Biotechnol.* **38**, 1274–1279 (2020). 33046875 10.1038/s41587-020-0703-0 PMC7642171

[71] Livak, K. J. & Schmittgen, T. D. Analysis of relative gene expression data using real-time quantitative PCR and the 2(-Delta Delta C(T)) method. *Methods* **25**, 402–408 (2001). 11846609 10.1006/meth.2001.1262

[72] White, B. et al. De novo annotation of the wheat pan-genome reveals complexity and diversity within the hexaploid wheat pan-transcriptome. Preprint at *bioRxiv*10.1101/2024.01.09.574802 (2024).

[73] Robinson, J. T. et al. Integrative genomics viewer. *Nat. Biotechnol.* **29**, 24–26 (2011). 21221095 10.1038/nbt.1754 PMC3346182

[74] Jumper, J. et al. Highly accurate protein structure prediction with AlphaFold. *Nature* **596**, 583–589 (2021). 34265844 10.1038/s41586-021-03819-2 PMC8371605

[75] Paysan-Lafosse, T. et al. InterPro in 2022. *Nucleic Acids Res.* **51**, D418–D427 (2023). 36350672 10.1093/nar/gkac993 PMC9825450

[76] The PyMol Molecular Graphics System v. 2.5. (Schrodinger LLC, 2021).

[77] Altschul, S. F., Gish, W., Miller, W., Myers, E. W. & Lipman, D. J. Basic local alignment search tool. *J. Mol. Biol.* **215**, 403–410 (1990). 2231712 10.1016/S0022-2836(05)80360-2

[78] Edgar, R. C. MUSCLE: multiple sequence alignment with high accuracy and high throughput. *Nucleic Acids Res.* **32**, 1792–1797 (2004). 15034147 10.1093/nar/gkh340 PMC390337

[79] Kalyaanamoorthy, S., Minh, B. Q., Wong, T. K. F., Von Haeseler, A. & Jermiin, L. S. ModelFinder: fast model selection for accurate phylogenetic estimates. *Nat. Methods* **14**, 587–589 (2017). 28481363 10.1038/nmeth.4285 PMC5453245

[80] Nguyen, L. T., Schmidt, H. A., Von Haeseler, A. & Minh, B. Q. IQ-TREE: a fast and effective stochastic algorithm for estimating maximum-likelihood phylogenies. *Mol. Biol. Evol.* **32**, 268–274 (2015). 25371430 10.1093/molbev/msu300 PMC4271533

[81] Hoang, D. T., Chernomor, O., Von Haeseler, A., Minh, B. Q. & Vinh, L. S. UFBoot2: improving the ultrafast bootstrap approximation. *Mol. Biol. Evol.* **35**, 518–522 (2018). 29077904 10.1093/molbev/msx281 PMC5850222

[82] Letunic, I. & Bork, P. Interactive Tree of Life (iTOL) v5: an online tool for phylogenetic tree display and annotation. *Nucleic Acids Res.* **49**, W293–W296 (2021). 33885785 10.1093/nar/gkab301 PMC8265157

[83] Hafeez, A. N. et al. Stb15 pathology data, *Zenodo*, https://zenodo.org/records/14515753 (2025).

[84] International Wheat Genome Sequencing Consortium Shifting the limits in wheat research and breeding using a fully annotated reference genome. *Science* **361**, eaar7191 (2018). 10.1126/science.aar7191 30115783

