# Supplementary material for: Septoria tritici blotch resistance gene Stb15 encodes a lectin receptor-like kinase
Source: Nat Plants. 2025 Mar 14;11(3):410–20. doi: 10.1038/s41477-025-01920-2 (PMC11928318; doi:10.1038/s41477-025-01920-2)
Supplement: Supplementary file 1 — Supplementary Figs. 1–9, Tables 1–17 and Text 1. [file 41477_2025_1920_MOESM1_ESM.pdf]

nature plants

Supplementary information

https://doi.org/10.1038/s41477-025-01920-2

Septoria tritici blotch resistance gene *Stb15* encodes a lectin receptor-like kinase

In the format provided by the authors and unedited

Supplementary Information: Table of Contents

Supplementary Fig. 1: Manhattan plot showing associations between SNPs in the Watkins landrace collection and damage (% maximum dAUDPC) in response to *Z. tritici* isolate IPO323.

Supplementary Fig. 2: Zoomed-in Manhattan plot of the 3A peak from the SNP-based GWAS with the IPO323 pycnidia phenotype, with a linkage disequilibrium heatmap.

Supplementary Fig. 3: Manhattan plot showing association of SNPs mapped to Chinese Spring with damage (% maximum dAUDPC) in response to *Z. tritici* isolate IPO88004.

Supplementary Fig. 4: A matrix of the distance in SNPs between Watkins accessions within the TraesCS6A02G078700 locus rendered as a heat map, with phenotype scores (pycnidia and damage) and major haplotype groups.

Supplementary Fig. 5: Manhattan plot of Watkins SNPs associated with IPO88004 pycnidia data, after removing lines carrying the functional allele of *Stb15* candidate TraesCS6A02G078700.

Supplementary Figure 6: pLDDT plot (A), pLDDT coloured structure (B), and predicted aligned error plot (C) for the highest confidence prediction of Arina *Stb15*.

Supplementary Fig. 7: Response of cv. Fielder stable T_2-stage transgenics and wheat control lines to inoculation with *Z. tritici* isolates IPO88004 (avirulent to *Stb15*) and IPO92006 (control virulent isolate), including mean pycnidia and damage scores.

Supplementary Fig. 8: Phylogenetic tree clade containing *Stb15* generated from a dataset of the top 30 BLAST protein hits to the Arina*LrFor* allele of *Stb15* (denoted Arina*LF* in tree) from 32 plant species with high-quality published genome assemblies. This clade features 16 Poaceae species.

Supplementary Fig. 9: Microsynteny of the *Stb15* mapping interval and adjacent genes. G-type lectin receptor-like kinase genes (magenta) represent *Stb15* and its orthologs.

Supplementary Table 1: REML variance components analysis of fixed terms for the linear mixed model analysis of logit pAUDPC scores from the Watkins 300 collection inoculated with IPO323, IPO88004 and IPO90012.

Supplementary Table 2: REML variance components analysis of fixed terms for the linear mixed model analysis of % maximum dAUDPC scores from the Watkins 300 collection inoculated with IPO323, IPO88004 and IPO90012.

Supplementary Table 3: Random effects of the linear mixed model fitted to logit pAUDPC and % maximum dAUDPC scores from the Watkins 300 collection inoculated with IPO323, IPO88004 and IPO90012.

Supplementary Table 4: Estimated logit pAUDPC and percentage of the maximum dAUDPC scores of the Watkins and wheat lines tested against IPO323 (avirulent to *Stb6*), IPO88004 (avirulent to *Stb15*) and IPO90012 (avirulent to *Stb11*), including mean response of lines to all isolates is provided.

Supplementary Table 5: Wheat lines included in each Septoria assay, including wheat lines whose genomes have been sequenced by the International Wheat Genome Sequencing Consortium or as part of the wheat pangenome project

Supplementary Table 6: Genes within the region on chromosome 6A associated with resistance to *Z. tritici* isolate IPO88004 and their function, with reasons for not selecting genes as *Stb15* candidates.

Supplementary Table 7: Pycnidia scores from Arraiano et al. (2009) that demonstratesthat resistance to IPO88004 does not correlate with IPO89011, and therefore that the 2BL resistance to IPO88004 is distinct from *Stb9* resistance to IPO89011.

Supplementary Table 8: REML analysis of a linear mixed model of pycnidium formation on Fielder transgenic T_2 lines containing *Stb15*, nulls and wheat varieties inoculated with *Z. tritici* isolates IPO88004 and IPO92006.

Supplementary Table 9: REML analysis of a linear mixed model of damage on Fielder transgenic T_2 lines containing *Stb15*, nulls and wheat varieties inoculated with *Z. tritici* isolates IPO88004 and IPO92006.

Supplementary Table 10: Mean values of logit(pAUDPC) for Fielder transgenic T_2 lines containing *Stb15,* nulls and wheat varieties inoculated with *Z. tritici* isolate IPO88004 estimated from the model presented in **Supplementary Table 8**. These data support the hypothesis of *Stb15*-dependent resistance to pycnidium formation by IPO88004, which is avirulent to *Stb15*.

Supplementary Table 11: Pairwise comparisons for the logit pAUDPC phenotype between genotype groups averaged over levels of Test.

Supplementary Table 12: Pairwise comparisons for the % max. dAUDPC phenotype between genotype groups averaged over levels of Test.

Supplementary Table 13: SNPs identified from the WatSeq alignment against Chinese Spring within *Stb6*.

Supplementary Table 14: SNPs identified from the WatSeq alignment against Chinese Spring within the TraesCS6A02G078700 (*Stb15*) locus.

Supplementary Table 15: KASP genotyping of *Stb15* in European wheat cultivars.

Supplementary Table 16: Genomes used for comparative analyses.

Supplementary Table 17: Protein and gene IDs of *Stb15* homologs in **Fig. 3c**.

Supplementary Text 1: Country and region abbreviations used in Figure 3.

Supplementary Figures

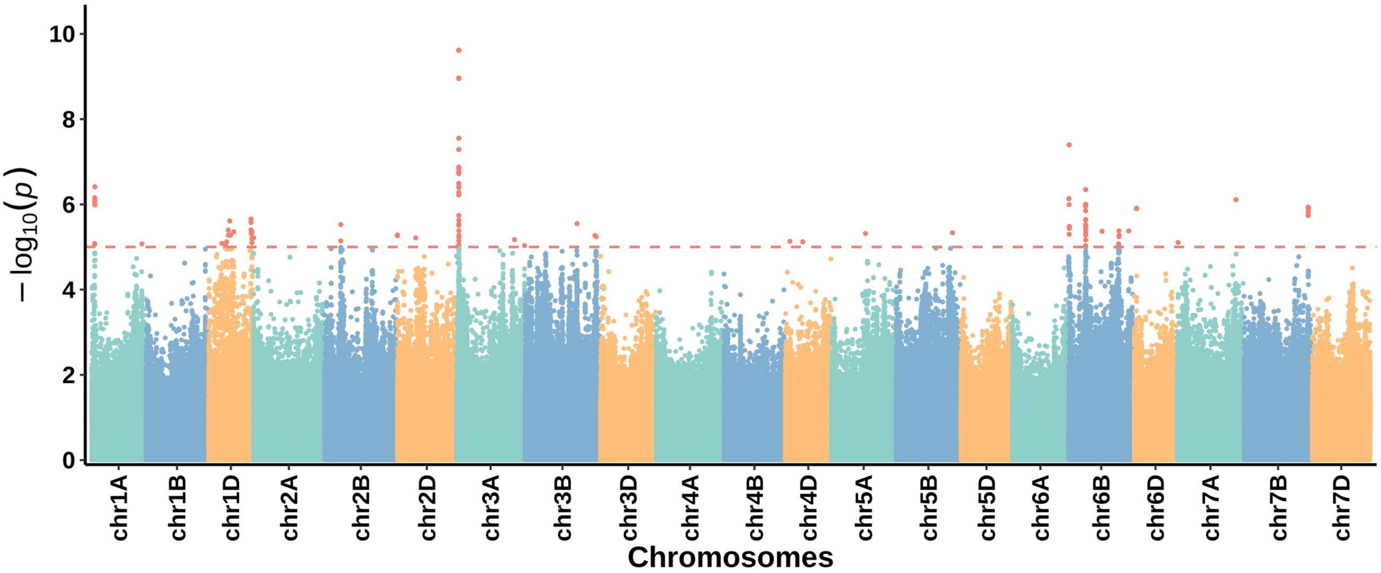

Supplementary Fig. 1: Manhattan plot showing associations between SNPs in the Watkins landrace collection and damage (% maximum dAUDPC) in response to *Z. tritici* isolate IPO323.

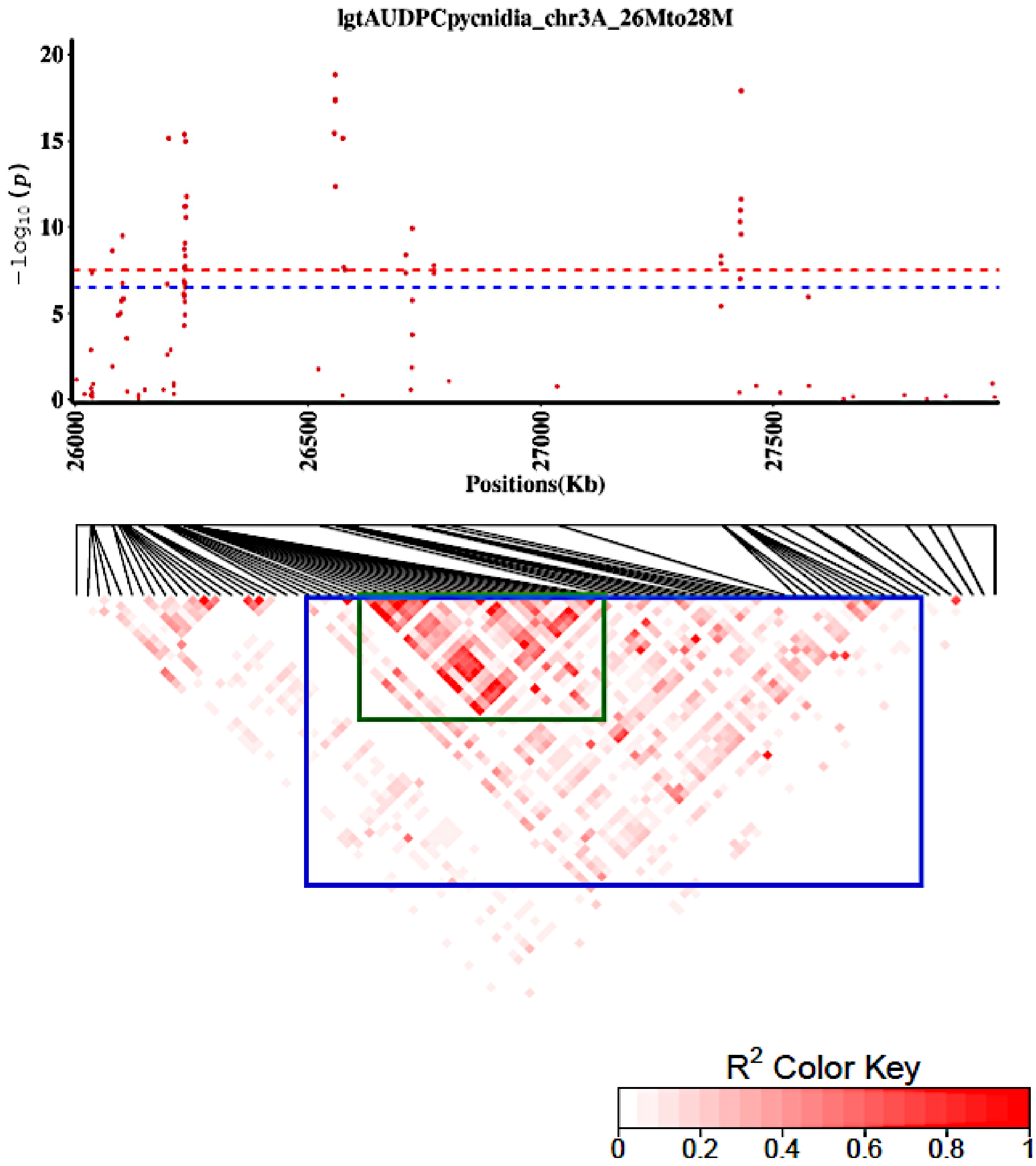

Supplementary Fig. 2: Zoomed-in Manhattan plot of the 3A peak from the SNP-based GWAS with the IPO323 pycnidia phenotype. The linkage disequilibrium heatmap is displayed below. Blue square from 26.10 to 27.50 Mb indicates the main area of the peak. Green square from 26,035,170 to 26,238,727 bp indicates the smaller haploblock within the blue square which is most highly associated with resistance.

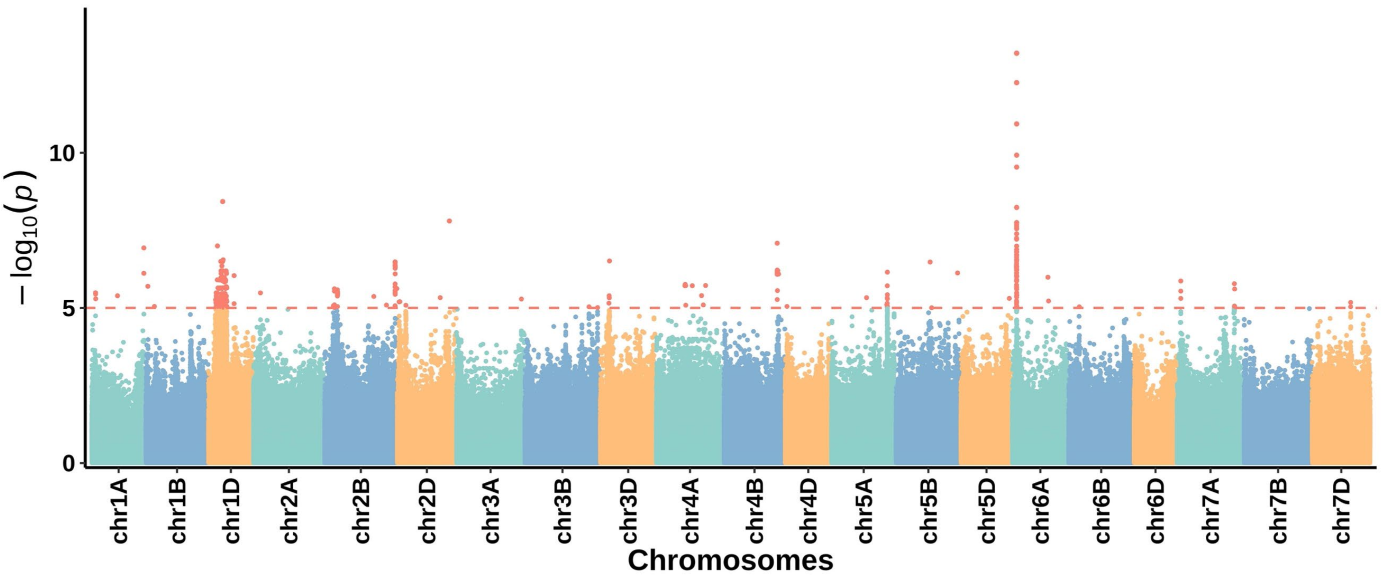

Supplementary Fig. 3: Manhattan plot showing association of SNPs mapped to Chinese Spring with damage (% maximum dAUDPC) in response to *Z. tritici* isolate IPO88004.

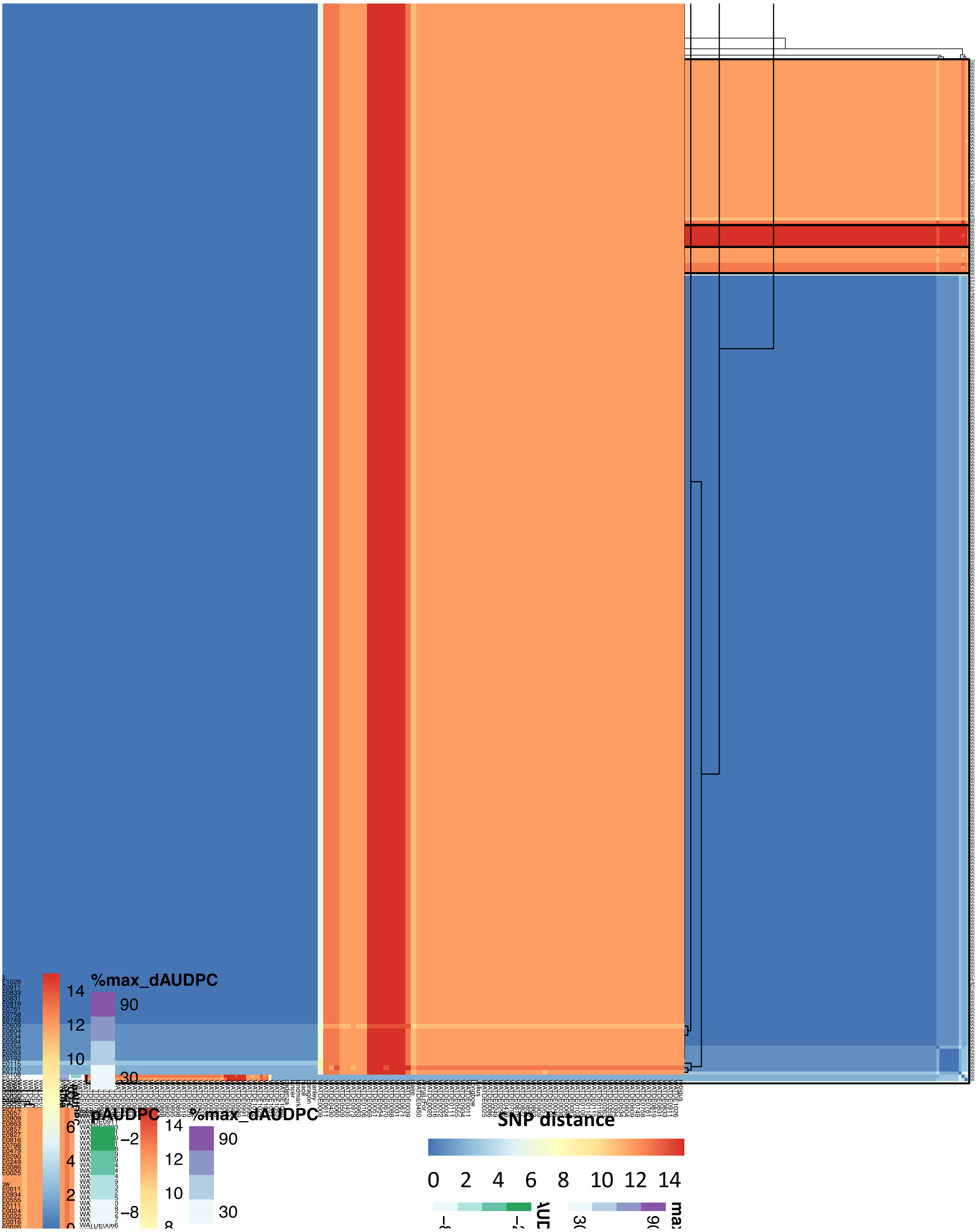

Supplementary Fig. 4: A matrix of the distance in SNPs between Watkins accessions within the TraesCS6A02G078700 locus rendered as a heat map. SNPs were called across a panel of 300 Watkins accessions in relation to landrace Chinese Spring (WatSeq consortium) and SNP distance calculated using a custom programme. Panel on the left shows phenotype scores: logit pAUDPC (pycnidia) in green and % max. dAUDPC (damage) in purple. Major haplotype groups are outlined in black and labelled with the names of key lines within those groups.

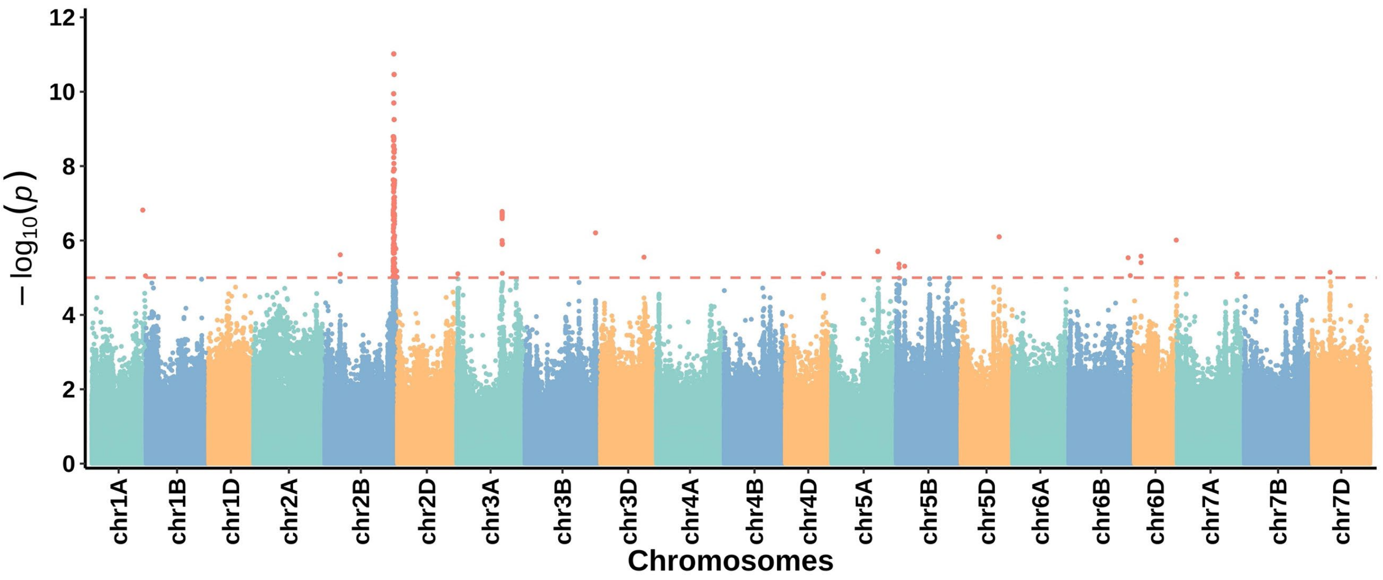

Supplementary Fig. 5: Manhattan plot of Watkins SNPs associated with IPO88004 pycnidia data, after removing lines carrying the functional allele of *Stb15* candidate TraesCS6A02G078700.

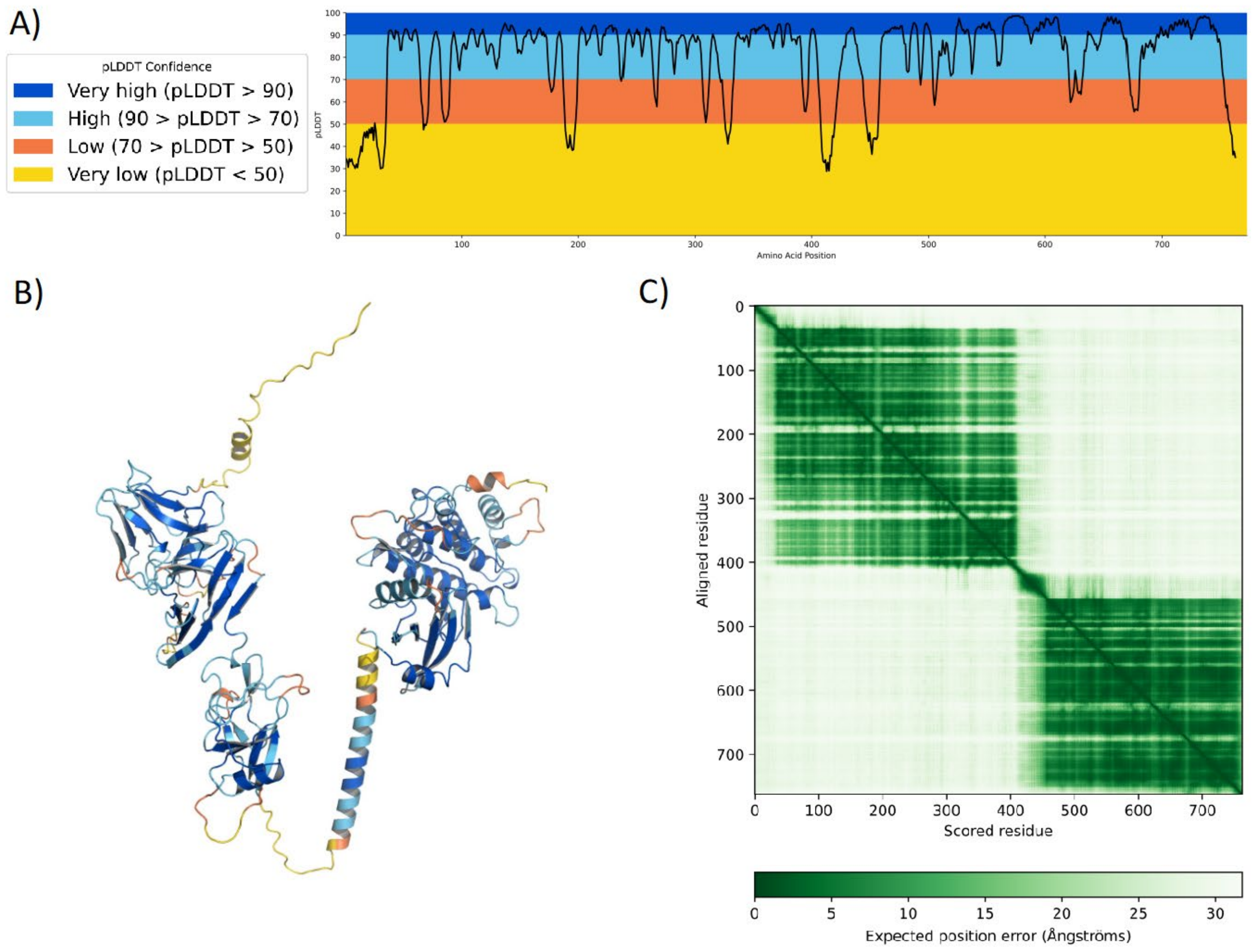

Supplementary Figure 6: pLDDT plot (A), pLDDT coloured structure (B), and predicted aligned error plot (C) for the highest confidence prediction of Arina Stb15 (Average pLDDT = 80.79).

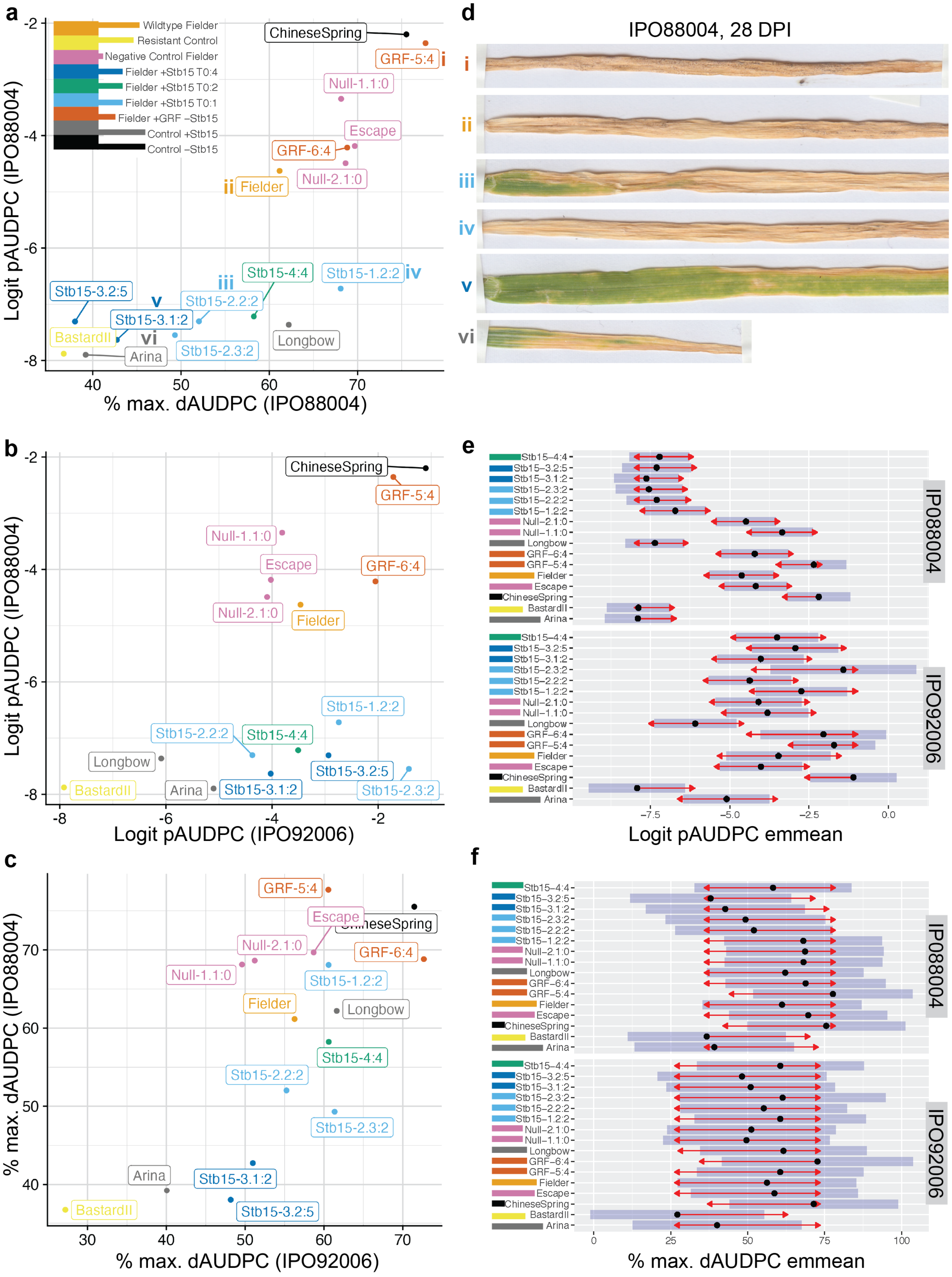

Supplementary Fig. 7: Response of cv. Fielder stable T_2-stage transgenics and wheat control lines to inoculation with *Z. tritici* isolates IPO88004 (avirulent to *Stb15*) and IPO92006 (control virulent isolate). The assay was repeated twice with ten replicates for IPO88004 and five for IPO92006 in each assay – a total of

up to 20 replicates for responses to IPO88004, with some exceptions where plants became damaged/died. Transgenic lines fall into four main groups: Fielder +*Stb15* with a T_0 copy number of 1, 2 or 4 and Fielder transformed with the same construct but without *Stb15* (including the GRF-GIF genes for improved transformation efficiency). The escape line underwent the transformation process but was not successfully transformed with the construct. Name codes for transgenic lines are described in **Supplementary Table 10**. **a-c**, Plots of means estimated from a linear mixed model (**Supplementary Tables 8-9**). Colours (key in panel **a**) correspond to groups of similar genotypes (*e.g.* transgenics with the same copy number of *Stb15* at the T_0 stage) and indicate purpose for inclusion of wheat lines (*e.g.* Arina is included as a resistant control that has *Stb15*). **a**, Means of pycnidia (logit pAUDPC) and damage (% of maximum possible dAUDPC) phenotypes to IPO88004 plotted against each other. Representative images from the second assay, labelled **i-vi**, for some lines are shown in panel **d**. The leaves shown were collected at 28 DPI, mounted on the same sheet of paper and scanned, followed by cropping of each leaf from the base upwards from the same original image to maintain relative sizes and consistent representation of the leaves. **b**, mean pycnidia scores for IPO88004 (y axis) and IPO92006 (x axis). **c**, mean damage scores for IPO88004 (y axis) and IPO92006 (x axis). **e-f**, estimated marginal means (emmeans) plots of pycnidia (**e**) and damage (**f**) scores indicating pairwise interactions between each genotype group, calculated from Tukey-adjusted emmeans (**Supplementary Table 10**). Black dots indicate emmeans for each line and blue boxes represent the confidence interval. Where red arrows overlap, the groups are not significantly statistically different from each other. *P*-values of pairwise comparisons are given in **Supplementary Tables 11-12**.

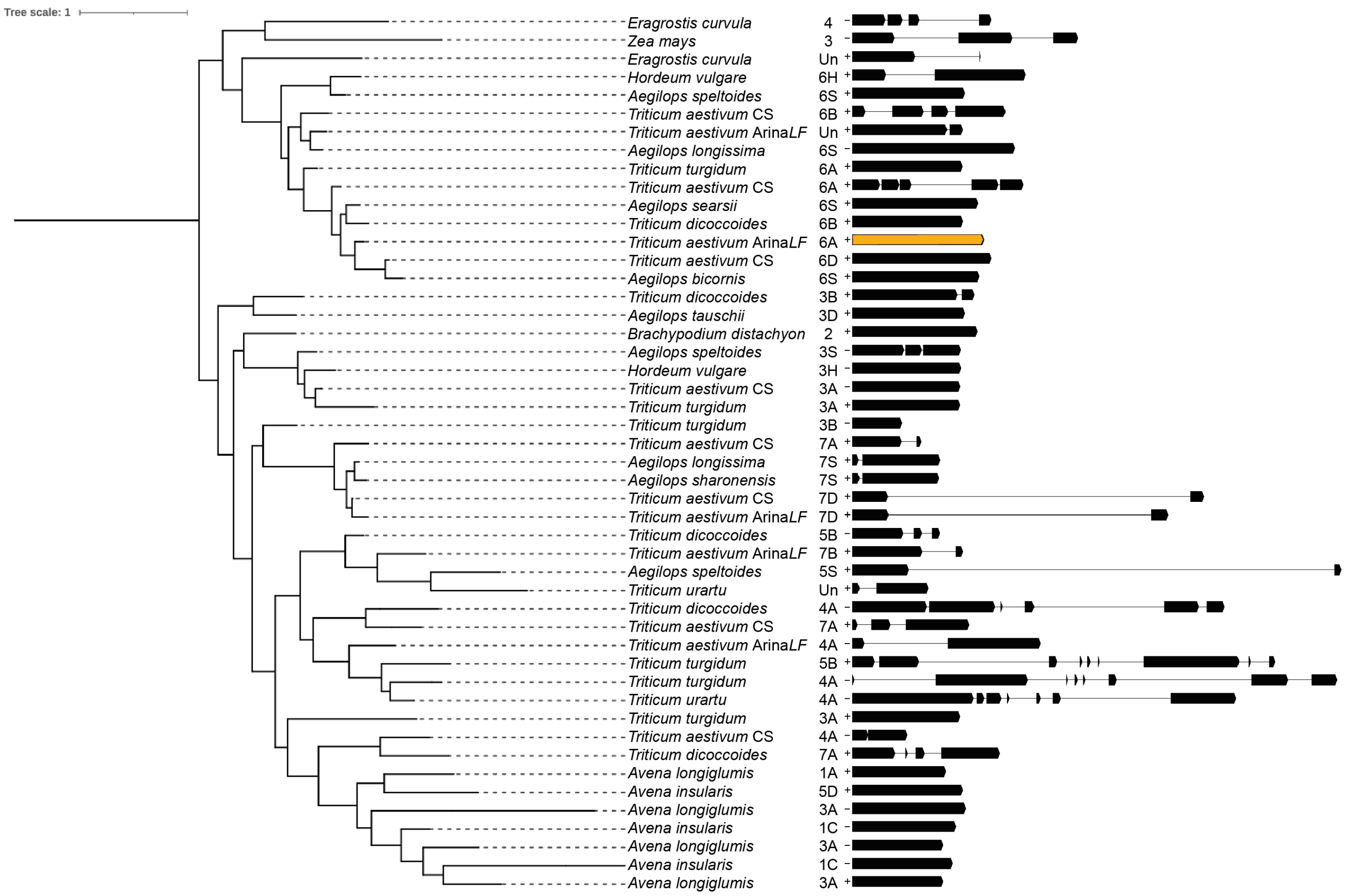

Supplementary Fig. 8: Phylogenetic tree clade containing *Stb15* generated from a dataset of the top 30 BLAST protein hits to the Arina*LrFor* allele of *Stb15* (denoted Arina*LF* in tree) from 32 plant species with high-quality published genome assemblies. This clade features 16 Poaceae species. Gene structures are given on the right based on annotation files for each genome. Arrows indicate exons whilst lines indicate introns. *Stb15* is highlighted in orange.

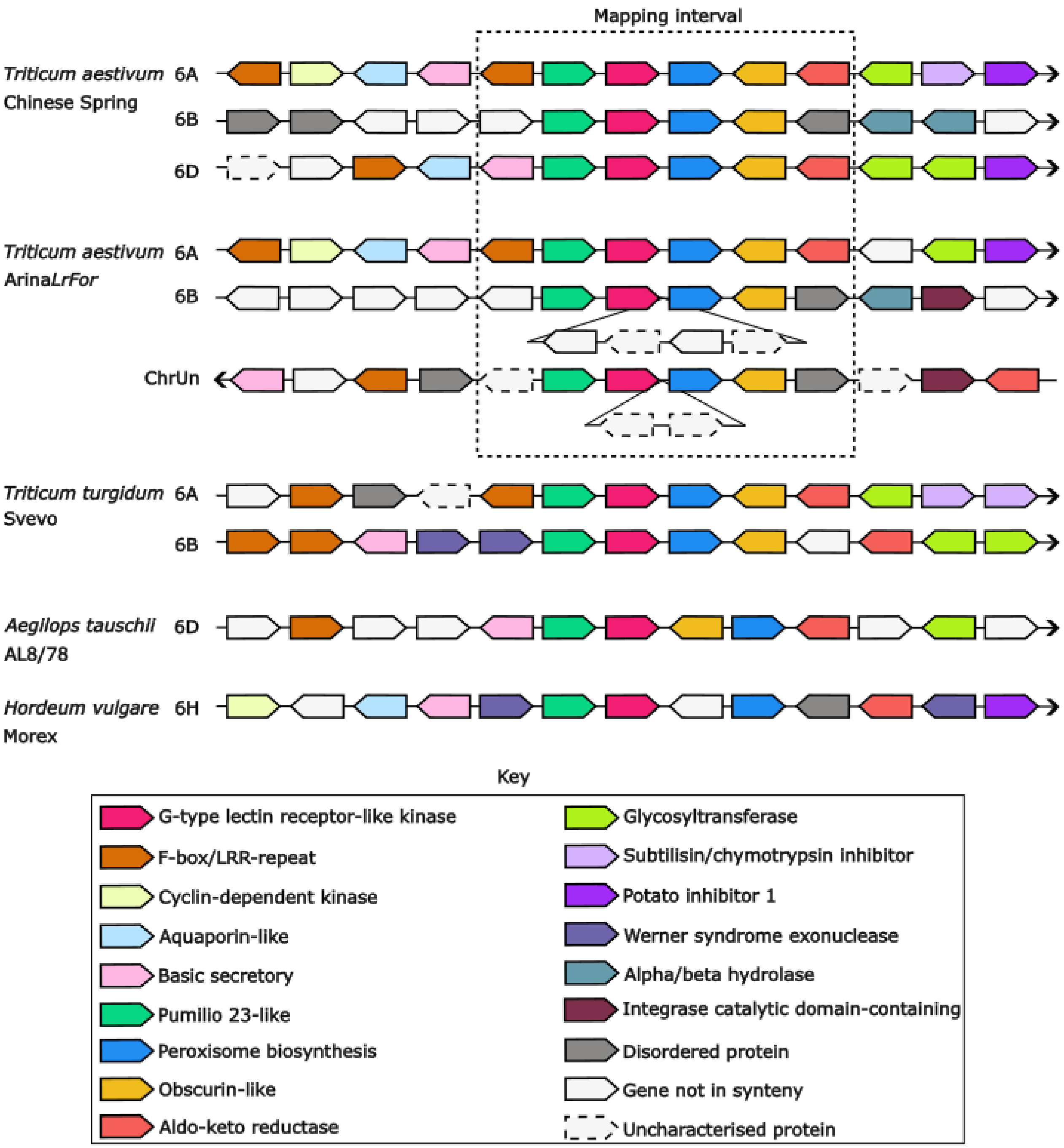

Supplementary Fig. 9: Microsynteny of the *Stb15* mapping interval and adjacent genes. G-type lectin receptor-like kinase genes (magenta) represent *Stb15* and its orthologs. The 99 kb mapping interval is indicated for *Triticum aestivum* lines Chinese Spring and Arina*LrFor*, which were included in the GWAS. Arina*LrFor* ChrUn contains a homolog of *Stb15* which is closely related to the *Aegilops longissima* and Chinese Spring 6B homologs; since a 6D homolog was not found, this chromosome is displayed. To allow better observation of the microsynteny, some proteins which are uncharacterised or not in synteny were collapsed for Arina*LrFor* chromosomes 6B and ChrUn. Directionality of each interval is indicated with an arrow; only ChrUn has been displayed in the reverse order for better alignment.

Supplementary Tables

Supplementary Table 1: REML variance components analysis of fixed terms for the linear mixed model analysis of logit pAUDPC scores from the Watkins 300 collection inoculated with IPO323, IPO88004 and IPO90012. Colons represent combinations of factors.

Term	Mean Square	Numerator DF	Denominator DF	F value	Pr(>F)
Isolate	21.45	2.00	3.10	8.53	0.05
Line	28.49	322.00	3539.20	11.34	<0.0001
Scorer	5.89	2.00	46.80	2.34	0.1
Isolate:Line	14.40	624.00	3535.20	5.73	<0.0001

Supplementary Table 2: REML variance components analysis of fixed terms for the linear mixed model analysis of % maximum dAUDPC scores from the Watkins 300 collection inoculated with IPO323, IPO88004 and IPO90012. Colons represent combinations of factors.

Term	Mean Square	Numerator DF	Denominator DF	F value	Pr(>F)
Isolate	607.48	2.00	3.00	12.46	0.03
Line	427.76	322.00	3523.50	8.77	<0.0001
Scorer	3.78	2.00	60.10	0.08	0.9
Isolate:Line	219.63	624.00	3502.20	4.51	<0.0001

Supplementary Table 3: Random effects of the linear mixed model fitted to logit pAUDPC and % maximum dAUDPC scores from the Watkins 300 collection inoculated with IPO323, IPO88004 and IPO90012. Colons represent combinations of factors.

	Logit pAUDPC		% max. dAUDPC	
Term	Variance	Std. Dev	Variance	Std. Dev
Isolate:Batch:Rep:Box:Tray			0.54	0.74
Isolate:Batch:Rep:Box	0.11	0.33	2.19	1.48
Isolate:Batch:Rep	0.01	0.12	0.00	0.00
Isolate:Batch	0.37	0.61	23.56	4.85
Residual	2.51	1.59	48.75	6.98

Supplementary Table 4: Estimated logit pAUDPC and percentage of the maximum dAUDPC scores of the Watkins and wheat lines tested against IPO323 (avirulent to *Stb6*), IPO88004 (avirulent to *Stb15*) and IPO90012 (avirulent to *Stb11*). Mean response of lines to all isolates is provided. Some wheat controls were only included in two assays so do not have a line mean estimate across all isolates, but a mean calculated from mean responses to the tested isolates is given in italics (this was not estimated via linear mixed modelling). Haplotypes of *Stb6* and *Stb15* inferred from WatSeq SNPs are also provided. For *Stb6*, haplotypes 1 and 2 are known resistant haplotypes whilst for *Stb15* A/R is known to be resistant and CS is susceptible. The WATDE/WatSeq column contains the names for each line that were used in the WatSeq resources employed in this study. See 'Stb15, Hafeez 2024, supplementary tables.pdf'.

Supplementary Table 5: Wheat lines included in each Septoria assay and reasons for their inclusion (yes = included). The top section of the table includes wheat lines whose genomes have been sequenced by IWGSC (International Wheat Genome Sequencing Consortium (IWGSC) et al., 2018) or as part of the wheat pangenome project (Walkowiak et al., 2020). Below are selections based on information from Arraiano & Brown (2006), Brown et al. (2015) and Chartrain et al. (2004). The bottom panel consists of cv. Fielder and results from this project.

Cultivar	IPO323	IPO88004	IPO90012	Reason for inclusion
Chinese Spring	Yes	Yes	Yes	Wheat reference genome
Arina*LrFor*	Yes	Yes	Yes	Wheat pangenome
Baj	Yes	Yes	Yes	Wheat cultivar
Cadenza	Yes	Yes	Yes	Wheat pangenome
CDC Landmark	Yes	Yes	Yes	Wheat pangenome
CDC Stanley	No	Yes	Yes	Wheat pangenome
Claire	No	Yes	Yes	Wheat pangenome
Kronos	No	Yes	Yes	Wheat pangenome
Jagger	No	Yes	Yes	Wheat pangenome
Julius	No	Yes	Yes	Wheat pangenome
Lancer	Yes	Yes	Yes	Wheat pangenome
Mace	No	Yes	Yes	Wheat pangenome
Norin 61	No	Yes	Yes	Wheat pangenome
Paragon	Yes	Yes	Yes	Wheat pangenome
Robigus	Yes	Yes	Yes	Wheat pangenome
SY Mattis	No	Yes	Yes	Wheat pangenome
Weebill	No	Yes	Yes	Wheat pangenome
Courtot	No	No	Yes	Susceptible to IPO90012
Flame	Yes	No	No	Resistant to IPO323
Longbow	Yes	Yes	Yes	Widely susceptible
Olaf	No	No	Yes	Resistant to IPO90012
Fielder	No	Yes	No	Susceptible to IPO88004, background of transgenics

Supplementary Table 6: Genes within the region on chromosome 6A associated with resistance to *Z. tritici* isolate IPO88004 and their function. Reasons for not selecting these genes as the *Stb15* candidate are given.

Annotation	Gene name	Start	Length (bp)	Protein	Reason for exclusion
TRAESCS6A02G078600	APUM23	48516680	5780	Pumilio homolog 23	Arina*LrFor* has the same genotype as Chinese Spring.
TRAESCS6A02G079000	S6PDH	48563464	3903	Aldo-ket-red domain-containing protein	Association of haplotypes with resistance phenotypes is not strong.
TRAESCS6A02G078800	PEX16	48552372	4331	Peroxisomal membrane protein PEX16	No SNPs in exons detected.
TRAESCS6A02G078900	SRK6	48556992	4149	Uncharacterised protein	Association of haplotypes with resistance phenotypes is not strong.
TRAESCS6A02G078700		48525265	3354	Receptor-like serine/threonine-protein kinase	**No reason to exclude** – clear association of the Arina*LrFor* haplotype group with resistance (**Supplementary Figure 4**).
TRAESCS6A02G078500		48509308	1890	Uncharacterised, LRR superfamily related domain	Arina*LrFor* has the same genotype as Chinese Spring.

Supplementary Table 7: Pycnidia scores from Arraiano et al. (2009) that demonstrate that resistance to IPO88004 does not correlate with IPO89011, and therefore that the 2BL resistance to IPO88004 is distinct from *Stb9* resistance to IPO89011. Emboldening and asterisks indicate specific resistance.

Cultivar	IPO88004	IPO89011	Conclusion
Sportsman	**1***	14	Resistant to IPO88004 but not to IPO89011
Selkirk	**0***	21	
Tipstaff	**1***	35	
Carstens VIII	**0***	36	
Maris Settler	**0***	30	
Courtot	67	**12***	Resistant to IPO89011 but not to IPO88004
Melbor	27	**1***	
Tonic	20	**5***	
Jena	47	**1***	
Soissons	45	**6***	
Plus 59 more cultivars resistant to IPO89011 but not IPO88004			

Supplementary Table 8: REML analysis of a linear mixed model of pycnidium formation on Fielder transgenic T_2 lines containing *Stb15*, nulls and wheat varieties inoculated with *Z. tritici* isolates IPO88004 and IPO92006. The variate analysed was logit-transformed area under the disease progress curve of pycnidial lesions (logit pAUDPC). The fixed effect model was Test * Isolate + Isolate + Cultivar whilst the random model was Test:Tray + Test:Tray:Rep (* and :, crossing and interaction operators).

The highly significant term for Isolate:Cultivar indicates strong evidence for isolate-specific variation in pycnidium formation, and thus for isolate-specific resistance to IPO88004, which is avirulent to *Stb15* (see Supplementary Figures 6b and 6e).

Fixed effects

Term	Numerator DF	Denominator DF	F statistic	P value
Test	1	12.48	4.2256	0.06
Isolate	1	13.14	32.6535	<0.0001
Cultivar	15	348.64	16.8164	<0.0001
Test:Isolate	1	12.48	15.0963	0.002
Isolate:Cultivar	15	348.64	4.5851	<0.0001

Random effects

**Term** [1]	**Variance component**	**Standard deviation**
Test:Tray:Rep	0.06336	0.2517
Test:Tray	0.30042	0.5481
Residual	3.71778	1.9282

[1] The Test:Variety term was omitted from the model because it had a small, negative variance component.

Supplementary Table 9: REML analysis of a linear mixed model of damage on Fielder transgenic T_2 lines containing *Stb15*, nulls and wheat varieties inoculated with *Z. tritici* isolates IPO88004 and IPO92006. The variate analysed was area under the disease progress curve (AUDPC) of damage on the leaf, including necrosis (dAUDPC). Fixed and random models were as in Supplementary Table 8.

As the Isolate:Cultivar term is not statistically significant, the hypothesis that there is no isolate-specific variation in foliar damage cannot be rejected (see Supplementary Figures 6c and 6f).

Fixed effects

Term	Numerator DF	Denominator DF	F statistic	P value
Test	1	23.44	0.0792	0.8
Isolate	1	12.97	0.3905	0.5
Cultivar	15	15.64	0.964	0.5
Test:Isolate	1	12.6	21.0253	0.0006
Isolate:Cultivar	15	30.97	1.215	0.3

Random effects

**Term**	**Variance component**	**Standard deviation**
Test:Variety	235.4	15.343
Test:Tray:Rep	11.38	3.374
Test:Tray	66.22	8.138
Residual	417.48	20.432

Supplementary Table 10: Mean values of logit(pAUDPC) for Fielder transgenic T_2 lines containing *Stb15,* nulls and wheat varieties inoculated with *Z. tritici* isolate IPO88004 estimated from the model presented in **Supplementary Table 8**. These data support the hypothesis of *Stb15*-dependent resistance to pycnidium formation by IPO88004, which is avirulent to *Stb15*. Transgenic lines have a number code of X.Y:Z which refers to the T_0 parent (X), T_1 parent (Y) and T_1 copy number (Z). *E.g.*, Stb15-2.2:2 and Stb15-2.1:0 came from the same T_0 parent but different T_1 plants with differing copy numbers. Copy number of T_2 plants is estimated from T_1 parents. 'Copy number GRF-GIF construct' refers to the copy number of the construct including the GRF-GIF genes for increased transformation efficiency, independent of the presence of *Stb15.*

Line	Genotype group	T_0 plant origin	T_0 copy number *Stb15*	T_0 copy number GRF-GIF construct	T_1 copy number *Stb15*	T_1 copy number GRF-GIF construct	Predicted T_2 copy number *Stb15*	Estimated mean logit pAUDPC (IPO88004)	SE	Estimated mean % pAUDPC (IPO88004)
**Fielder**	Wiltype Fielder							-7.90	0.53	0.0
**Escape**	Negative Control Fielder							-4.18	0.50	1.5
**Null-1.1:0**	Negative Control Fielder	1	1	1	0	0	0	-3.35	0.49	3.4
**Null-2.1:0**	Negative Control Fielder	2	1	1	0	0	0	-4.49	0.48	1.1
**Stb15-1.2:2**	Fielder +Stb15 T0:1	1	1	1	2	2	2	-6.72	0.49	0.1
**Stb15-2.2:2**	Fielder +Stb15 T0:1	2	1	1	2	2	2	-7.30	0.48	0.0
**Stb15-2.3:2**	Fielder +Stb15 T0:1	2	1	1	2	2	2	-7.55	0.53	0.0
**Stb15-3.1:2**	Fielder +Stb15 T0:4	3	4	4	2	2	0-4	-7.63	0.52	0.0
**Stb15-3.2:5**	Fielder +Stb15 T0:4	3	4	4	5	5	2-6	-7.31	0.55	0.0
**Stb15-3.3:7**	Fielder +Stb15 T0:4	3	4	4	7	7	6-8	-	-	-
**Stb15-4:4**	Fielder +Stb15 T0:4	4	2	2	4	4	4	-7.22	0.48	0.0
**GRF-5:4**	Fielder +GRF -Stb15	5	0	2	0	4	0	-1.71	0.66	15.3
**GRF-6:4**	Fielder +GRF -Stb15	6	0	2	0	4	0	-2.05	1.00	11.4
**Arina**	Control +Stb15							-5.10	0.69	0.6
**Longbow**	Control +Stb15							-6.09	0.66	0.2
**BastardII**	Resistant Control							-7.92	0.77	0.0
**Chinese Spring**	Control -Stb15							-1.10	0.69	25.0

Supplementary Table 11: Pairwise comparisons for the logit pAUDPC phenotype between genotype groups averaged over levels of Test. Tukey post-hoc adjustment was applied and the emmeans package in R used to calculate comparisons. Comparisons which show that pycnidia scores with IPO88004 of Fielder +Stb15 at all tested copy number groups is significantly different from the most comparable control, Stb15 +GRF -Stb15, are indicated in red. These comparisons are also indicated for IPO92006. The comparisons indicate that there is *Stb15*-dependent resistance to pycnidium formation by IPO88004, which is avirulent to *Stb15*. See 'Stb15, Hafeez 2024, supplementary tables.pdf'.

Supplementary Table 12: Pairwise comparisons for the % max. dAUDPC phenotype between genotype groups averaged over levels of Test. Tukey post-hoc adjustment was applied and the emmeans package in R used to calculate comparisons. Comparisons coloured in red in Supplementary Table 11 are highlighted here to allow cross-reference. See 'Stb15, Hafeez 2024, supplementary tables.pdf'.

Supplementary Table 13: SNPs identified from the WatSeq alignment against Chinese Spring within *Stb6*.

Genomic position	Chinese Spring allele	Alternative allele
26,200,567	T	C
26,200,618	C	G
26,200,653	A	G
26,200,659	G	A
26,201,022	A	`G
26,201,037	C	T
26,201,046	T	C
26,201,734	G	A
26,202,969	G	A
26,203,022	A	G
26,203,076	A	T
26,203,115	A	G
26,203,148	C	A
26,203,155	G	T
26,203,192	G	T
26,203,263	C	T
26,203,264	A	G
26,203,430	C	T
26,205,300	A	T
26,205,344	T	C
26,206,172	T	A
26,206,287	T	G

Supplementary Table 14: SNPs identified from the WatSeq alignment against Chinese Spring within the TraesCS6A02G078700 (*Stb15*) locus. SNP genotype for each haplotype defined from **Supplementary Figure 4** is given. Robigus has the same genotype as Arina/Arina*LrFor* at this locus (based on wheat pangenome assemblies) but due to some areas of low read coverage there were missing datapoints in this locus for Arina*LrFor*, resulting in differences in SNP distance between Robigus and Arina*LrFor*. The more complete SNP dataset for Robigus can therefore be referred to here as the reference for the functional resistant allele of *Stb15.* The 'SNP distance from' columns indicate the number of SNPs which differentiate the haplotype groups in the leftmost column from either Chinese Spring or Arina*LrFor*.

		SNP distance from		SNP position chr6A_part1:4852...														
				1	2	3	4	5	6	7	8	9	10	11	12	13	14	15
**Haplotype**	**No.**	**Chinese Spring**	**Arina*LrFor***	5467	5473	5514	5805	5907	5922	5923	5925	6158	6193	7920	8004	8102	8609	8693
Chinese Spring	250	0-6	11-12	C	C	C	A	C	C	T	A	C	A	G	G	C	C	T
Robigus	51	11-12	0-7	A	G	C	A	A	A	C	G	T	C	A	T	A	C	C
Watkins 1	7	15	11	A	G	C	.	.	.	.	.	.	.	A	T	A	T	C
Watkins 2	7	12	10-11	A	G	T	G	C	C	T	G	.	.	A	T	A	T	C
				EXON 1				EXON 2						EXON 4			EXON 5	

Supplementary Table 15: KASP genotyping of *Stb15* in European wheat cultivars. See 'Stb15, Hafeez 2024, supplementary tables.pdf'.

Supplementary Table 16: Genomes used for comparative analyses. See 'Stb15, Hafeez 2024, supplementary tables.pdf'.

Supplementary Table 17: Protein and gene IDs of *Stb15* homologs in **Fig. 3c**. See 'Stb15, Hafeez 2024, supplementary tables.pdf'.

Supplementary Text 1

Country and region abbreviations used in Figure 3.

AFG = Afghanistan, ALB = Albania, ALG = Algeria, ARM = Armenia, AUS = Australia, AZE = Azerbaijan, B&H = Bosnia & Herzegovina, BUL = Bulgaria, CHI = China, CI = Canary Islands, CRO = Croatia, CYP = Cyprus, EGY = Egypt, ETH = Ethiopia, FRA = France, FIN = Finland, GEO = Georgia, GRE = Greece, HUN = Hungary, IND = India, IRN = Iran, IRQ = Iraq, ITA = Italy, KAZ = Kazakhstan, LEB = Lebanon, MOR = Morocco, MYA = Myanmar, NM = North Macedonia, PAK = Pakistan, PAL = Palestine, POL = Poland, POR = Portugal, ROM = Romania, RUS = Russia, SER = Serbia, SPA = Spain, SYR = Syria, TKM = Turkmenistan, TUN = Tunisia, TUR = Turkey, UKR = Ukraine, UK = United Kingdom.
