# Supplementary material for: Septoria tritici blotch resistance gene Stb15 encodes a lectin receptor-like kinase
Source: Nat Plants. 2025 Mar 14;11(3):410–20. doi: 10.1038/s41477-025-01920-2 (PMC11928318; doi:10.1038/s41477-025-01920-2)
Supplement: Supplementary file 2 — Reporting Summary [file 41477_2025_1920_MOESM2_ESM.pdf]

nature portfolio

Corresponding author(s): James Brown, Brande Wulff

Last updated by author(s): 18/12/2024

Reporting Summary

Nature Portfolio wishes to improve the reproducibility of the work that we publish. This form provides structure for consistency and transparency in reporting. For further information on Nature Portfolio policies, see our Editorial Policies and the Editorial Policy Checklist.

Statistics

For all statistical analyses, confirm that the following items are present in the figure legend, table legend, main text, or Methods section.

n/a	Confirmed	
☐	☒	The exact sample size (n) for each experimental group/condition, given as a discrete number and unit of measurement
☐	☒	A statement on whether measurements were taken from distinct samples or whether the same sample was measured repeatedly
☐	☒	The statistical test(s) used AND whether they are one- or two-sided<br>*Only common tests should be described solely by name; describe more complex techniques in the Methods section.*
☐	☒	A description of all covariates tested
☐	☒	A description of any assumptions or corrections, such as tests of normality and adjustment for multiple comparisons
☐	☒	A full description of the statistical parameters including central tendency (e.g. means) or other basic estimates (e.g. regression coefficient) AND variation (e.g. standard deviation) or associated estimates of uncertainty (e.g. confidence intervals)
☐	☒	For null hypothesis testing, the test statistic (e.g. F, t, r) with confidence intervals, effect sizes, degrees of freedom and P value noted<br>*Give P values as exact values whenever suitable.*
☒	☐	For Bayesian analysis, information on the choice of priors and Markov chain Monte Carlo settings
☐	☒	For hierarchical and complex designs, identification of the appropriate level for tests and full reporting of outcomes
☒	☐	Estimates of effect sizes (e.g. Cohen's d, Pearson's r), indicating how they were calculated

Our web collection on statistics for biologists contains articles on many of the points above.

Software and code

Policy information about availability of computer code

Data collection	Leaf scans were measured by Canon IJ Scan Utility2. SNP data are available on GrassRoots, a public database at the Earlham Institute (see Cheng et al. 2024, Nature: https://doi.org/10.1038/s41586-024-07682-9).
Data analysis	The following software was used as described in the Methods section of the paper: Python 3 for a custom script to identify haplotypes of candidate Stb genes at the 6AS locus (https://github.com/ambernhafeez/VCFparse_distance.git); IGV version 2.14.0, BLAST version 2.13.0, MUSCLE version 3.8.31 and Geneious version 2022.2.2 for genomic analysis; AlphaFold version 2.3.2, Interproscan online server, PyMol version 2.5.2 and a custom script, AlphaFold Analyser version 2.0.0 (https://github.com/Orpowell/alphafold-analyser) for protein modelling, annotation and visualisation; IQ-TREE version 1.6.10 and iTOL version 6 for phylogenetics; GEMMA version 0.98.1 for GWAS-QTL analysis; Gendex for experimental design; and R version 4.2.2 and packages for analysis of pathology and other data: lmerTest, emmeans, ggplot2, cowplot, ggmap, vcfR, vegan, pheatmap.<br><br>Software for SNP mapping and GWAS-QTL mapping was reported previously (see Cheng et al. 2024, Nature: https://doi.org/10.1038/s41586-024-07682-9: https://github.com/ShifengCHENG-Laboratory/WWWG2B and https://github.com/JIC-CSB/WatSeqAnalysis/).

For manuscripts utilizing custom algorithms or software that are central to the research but not yet described in published literature, software must be made available to editors and reviewers. We strongly encourage code deposition in a community repository (e.g. GitHub). See the Nature Portfolio guidelines for submitting code & software for further information.

Data

Policy information about availability of data

All manuscripts must include a data availability statement. This statement should provide the following information, where applicable:

- Accession codes, unique identifiers, or web links for publicly available datasets
- A description of any restrictions on data availability
- For clinical datasets or third party data, please ensure that the statement adheres to our policy

SNP data5 are available at GrassRoots (https://opendata.earlham.ac.uk/wheat/under_license/toronto/WatSeq_2023-09-15_landrace_modern_Variation_Data/WatSeq_VCF_ChineseSpringRefSeqv1.0/). Pathology data are available at https://doi.org/10.5281/zenodo.14515753.. The Stb15 sequence has been reported with an annotated transcript on Ensembl Plants (TraesARI6A03G03215890.1).

Research involving human participants, their data, or biological material

Policy information about studies with human participants or human data. See also policy information about sex, gender (identity/presentation), and sexual orientation and race, ethnicity and racism.

Reporting on sex and gender	n/a
Reporting on race, ethnicity, or other socially relevant groupings	n/a
Population characteristics	n/a
Recruitment	n/a
Ethics oversight	n/a

Note that full information on the approval of the study protocol must also be provided in the manuscript.

Field-specific reporting

Please select the one below that is the best fit for your research. If you are not sure, read the appropriate sections before making your selection.

☒ Life sciences ☐ Behavioural & social sciences ☐ Ecological, evolutionary & environmental sciences

For a reference copy of the document with all sections, see nature.com/documents/nr-reporting-summary-flat.pdf

Life sciences study design

All studies must disclose on these points even when the disclosure is negative.

Sample size	Sample size in pathology experiments was based on extensive previous experience with this fungal disease.
Data exclusions	No data were excluded.
Replication	All pathology experiments were done in multiple replicates, as described in the Methods section.
Randomization	The large pathology experiment with 300+ wheat lines in the UK was done with an alpha-lattice design using random allocation of lines to blocks and randomisation within blocks. Other pathology experiments were done with randomised complete block designs.
Blinding	Disease was scored blind, without reference to the accession name or genotype.

Reporting for specific materials, systems and methods

We require information from authors about some types of materials, experimental systems and methods used in many studies. Here, indicate whether each material, system or method listed is relevant to your study. If you are not sure if a list item applies to your research, read the appropriate section before selecting a response.

Materials & experimental systems

n/a	Involved in the study
☒	☐ Antibodies
☒	☐ Eukaryotic cell lines
☒	☐ Palaeontology and archaeology
☒	☐ Animals and other organisms
☒	☐ Clinical data
☒	☐ Dual use research of concern
☐	☒ Plants

Methods

n/a	Involved in the study
☒	☐ ChIP-seq
☒	☐ Flow cytometry
☒	☐ MRI-based neuroimaging

Plants

Seed stocks

Seeds of wheat cultivar ArinaLrFor (PANG0001) and the Stb15 transgenic lines in the background of cv Fielder are available from the Germplasm Resources Unit, John Innes Centre, Norwich, UK (https://www.jic.ac.uk/research-impact/germplasm-resource-unit/). Seed of the mutagenized Arina population were supplied by Prof Dr Beat Keller, Zurich.

Novel plant genotypes

Methods for producing transgenic lines of cv Fielder are reported in the Methods section of this paper.

Authentication

Methods for authenticating the transgenic lines of cv Fielder are reported in the Methods section of this paper.
